# Progressive Microstructural Deterioration Dictates Evolving Biomechanical Dysfunction in the Marfan Aorta

**DOI:** 10.3389/fcvm.2021.800730

**Published:** 2021-12-16

**Authors:** Cristina Cavinato, Minghao Chen, Dar Weiss, Maria Jesús Ruiz-Rodríguez, Martin A. Schwartz, Jay D. Humphrey

**Affiliations:** ^1^Department of Biomedical Engineering, Yale University, New Haven, CT, United States; ^2^Cardiovascular Research Center and Department of Internal Medicine (Cardiology), Yale School of Medicine, New Haven, CT, United States; ^3^Centro Nacional de Investigaciones Cardiovasculares (CNIC) and Centro de Investigación Biomédica en Red de Enfermedades Cardiovasculares (CIBERCV), Madrid, Spain; ^4^Vascular Biology and Therapeutics Program, Yale School of Medicine, New Haven, CT, United States

**Keywords:** elastic fibers, fibrillin-1, collagen, Marfan syndrome, stiffness

## Abstract

Medial deterioration leading to thoracic aortic aneurysms arises from multiple causes, chief among them mutations to the gene that encodes fibrillin-1 and leads to Marfan syndrome. Fibrillin-1 microfibrils associate with elastin to form elastic fibers, which are essential structural, functional, and instructional components of the normal aortic wall. Compromised elastic fibers adversely impact overall structural integrity and alter smooth muscle cell phenotype. Despite significant progress in characterizing clinical, histopathological, and mechanical aspects of fibrillin-1 related aortopathies, a direct correlation between the progression of microstructural defects and the associated mechanical properties that dictate aortic functionality remains wanting. In this paper, age-matched wild-type, *Fbn1*^*C*1041*G*/+^, and *Fbn1*^*mgR*/*mgR*^ mouse models were selected to represent three stages of increasing severity of the Marfan aortic phenotype. *Ex vivo* multiphoton imaging and biaxial mechanical testing of the ascending and descending thoracic aorta under physiological loading conditions demonstrated that elastic fiber defects, collagen fiber remodeling, and cell reorganization increase with increasing dilatation. Three-dimensional microstructural characterization further revealed radial patterns of medial degeneration that become more uniform with increasing dilatation while correlating strongly with increased circumferential material stiffness and decreased elastic energy storage, both of which comprise aortic functionality.

## Introduction

Fibrillin-1 is an elastin-associated glycoprotein that plays important structural and instructional roles within the aortic wall (1), among other tissues. In particular, fibrillin-1 appears to contribute significantly to the long-term stability of elastic fibers (2), which have a half-life on the order of decades (3), and to endow the aortic wall with much of its resilience (4); it also regulates the bioavailability of transforming growth factor-beta (TGFβ) by sequestering latent TGFβ binding proteins within the extracellular matrix (5). TGFβ similarly plays many important roles in aortic biology, including stimulating matrix turnover (6) and modulating the actomyosin activity that contributes to mechano-sensing and mechano-regulation of the extracellular matrix (7). Multiple mouse models of fibrillin-1 deficiency have been used to study roles of this key glycoprotein on aortic structure, function, and disease progression. Among others, Ramirez and colleagues compared geometric, microstructural, and mechanical metrics in mice haploinsufficient for either elastin (*Eln*^+/−^) or fibrillin-1 (*Fbn1*^+/−^) and found both differential and complementary roles of these two key components of elastic fibers (8). Faury and colleagues reported, using a mouse model that expresses about 60% of normal fibrillin-1 (*Fbn1*^*mgΔ*/+^), that compromised fibrillin-1 leads to a progressive phenotype characterized by disrupted elastic lamellae, increased inter-lamellar distances, dispersed collagen fibers, and an overall aortic dilatation and stiffening (9). In neither case, however, was there a direct correlation between the microstructural defects and detailed metrics of the mechanical properties.

The importance of understanding roles of fibrillin-1 in aortic biology and mechanics is underscored further by the development of proximal aortic aneurysms, dissections, and rupture in Marfan syndrome, which results from mutations to the gene (*FBN1*) that encodes fibrillin-1 (10). Two particular mouse models have found considerable utility in studying the aortic phenotype in Marfan syndrome: the hypomorphic *Fbn1*^*mgR*/*mgR*^ mouse (expressing ~15% of the normal fibrillin-1) (2), which presents a severe Marfan-like phenotype including dilatation and rupture early in maturity, and the *Fbn1*^*C*1041*G*/+^ knock-in mouse (mimicking a specific human mutation) (11), which experiences a less-severe, slowly developing phenotype over the first year of life. Again, however, a correlation of the progressive deterioration of the aortic wall and the associated biomechanical properties that dictate aortic functionality has remained wanting. The two-fold goal of this work is to present detailed microstructural analyses of the aortic wall as a function of dilatation in the *Fbn1*^*C*1041*G*/+^ (a.k.a. *Fbn1*^*C*1039*G*/+^, but denoted herein as C1041G) and *Fbn1*^*mgR*/*mgR*^ (denoted herein as mgR) mouse models and to correlate the altered microstructural metrics with biaxial characterizations of wall properties.

## Results

**Figure 1:**
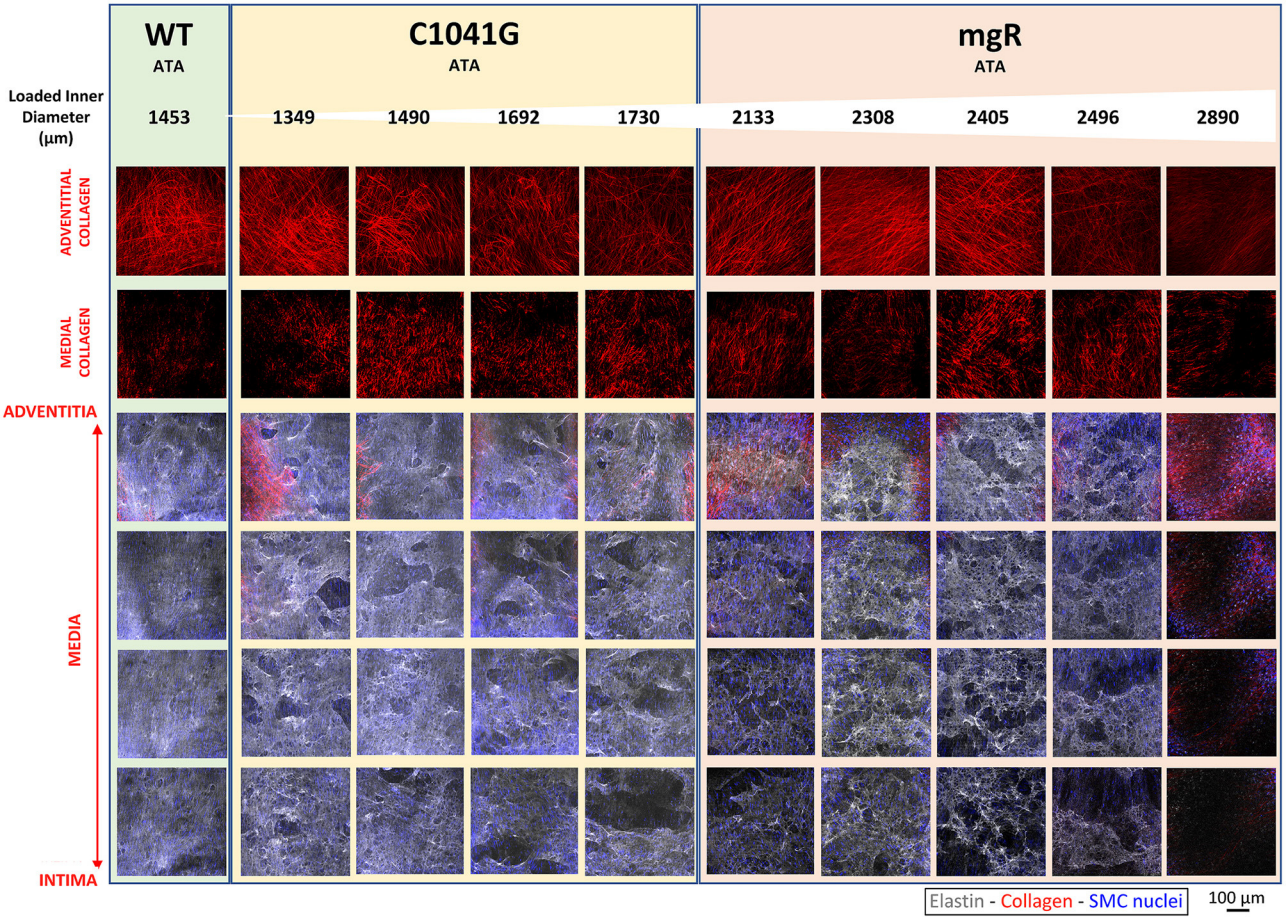
Mosaic of images from multiphoton microscopy of the ascending thoracic aorta (ATA) from 10 representative mice: one of the five wild-type (WT) controls plus four of the seven *Fbn1*^*C*1041*G*/+^ (C1041G) and five of the six *Fbn1*^*mgR*/*mgR*^ (mgR) Marfan mice that were imaged. Note that the images were collected *ex vivo* under *in vivo* relevant diastolic conditions, namely, at an 80 mmHg distending pressure and specimen-specific *in vivo* value of axial stretch. From top-to-bottom, adventitial then medial fibrillar collagen (red, via second harmonic generation) and four equally spaced medial elastin volumes (gray, two-photon fluorescence images), the latter with superimposed cell nuclei (blue). These representative images are ordered from left-to-right by increasing aortic diameter in the nine Marfan mice. Note the strong tendency of increased elastic fiber damage as a function of increasing dilatation and from the inner to outer medial layers. See Supplementary Figure 1A for similar results for three representative descending thoracic aortas, one per genotype. See also Supplementary Figure 1B for medial images without superimposed cell nuclei.

*Microstructural deterioration associates with increasing diameter*. It has long been known that mutations to the gene that encodes fibrillin-1 results in compromised elastic fiber integrity, most often observed as breaks in or fragments of the elastic lamellae in standard histology. Effects on collagen fibers are also noted frequently, though also descriptively. Representative images of adventitial and medial collagen fibers (second harmonic generation) and multiple layers of elastic fibers (two-photon fluorescence) through the entire depth of the ascending aortic wall for one representative wild-type (WT) control and nine Marfan mice of the same post-natal age (~P60) are compared in Figure 1, each while maintained *ex vivo* under diastolic biaxial loading conditions. When the Marfan aortas are ordered by increasing diameter (left-to-right in Figure 1), it is evident that defects to collagen and especially elastic fibers tend to increase with increasing aortic dilatation, and that defects to the elastic fibers initially tend to be greater within the inner layers of the media but to extend to outer layers with increasing dilatation, presenting in all cases as voids rather than simple linear breaks. Similar microstructures were observed for the descending thoracic aorta, which exhibits a milder phenotype in both mouse models (Supplementary Figure 1A). Also see Supplementary Figure 1B for an enlarged view of the elastic lamellar architecture in all three genotypes and both regions.

**Figure 2:**
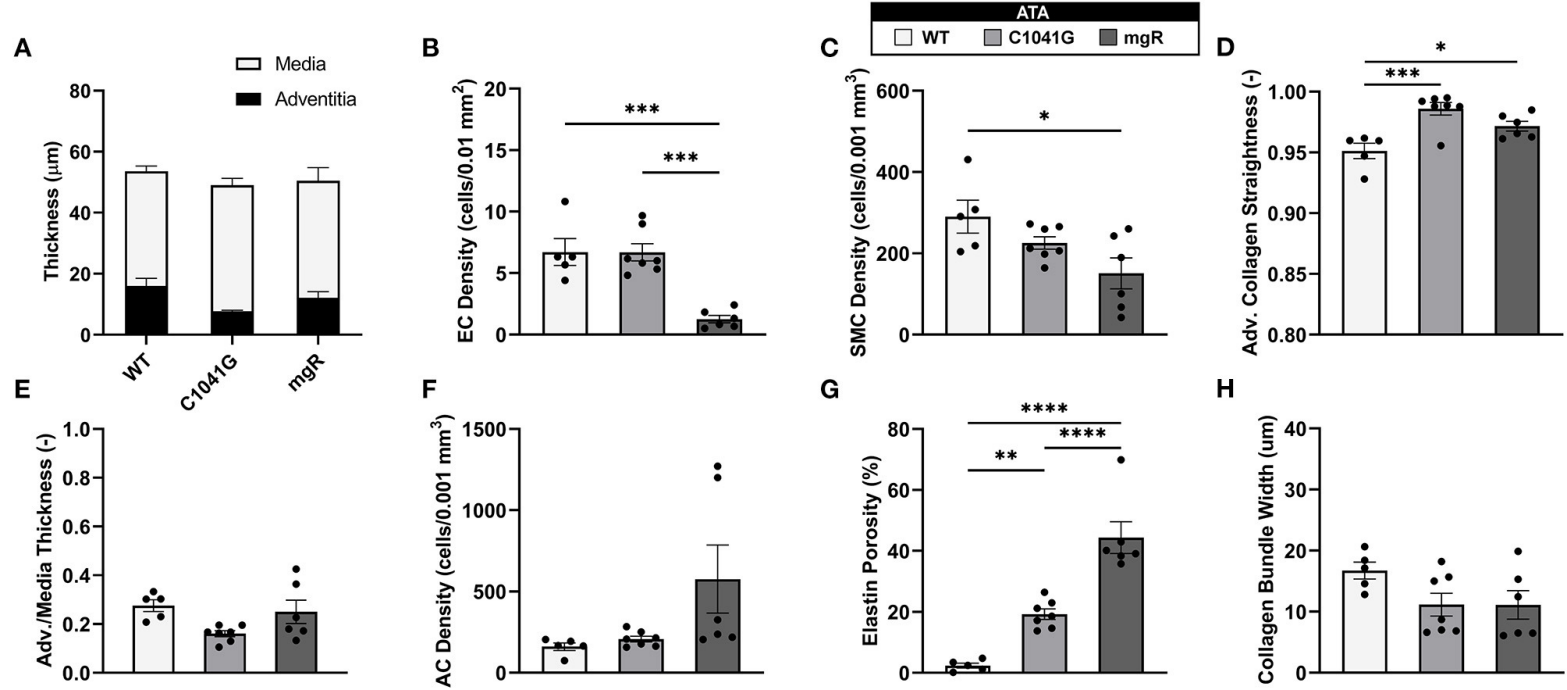
Multiple microstructural metrics for the ascending thoracic aorta (ATA) plotted as a function of genotype: WT (*n* = 5), C1041G (*n* = 7), and mgR (*n* = 6). Included are **(A)** medial and adventitial thicknesses, and **(E)** their ratio, plus layer-specific densities of **(B)** endothelial cells, **(C)** smooth muscle cells, and **(F)** adventitial cells based on cell nuclei and associated areas or volumes as well as **(G)** elastin porosity and **(D,H)** two measures of collagen fiber structure (straightness and fiber bundle width, respectively). Of particular note, smooth muscle cell density decreased while elastin porosity increased with increasing aortic diameter, that is, from WT to C1041G to mgR. Data are shown as mean ± SEM, with *p < 0.05, **p < 0.01, ***p < 0.001, and ****p < 0.0001. See Supplementary Figure 2 for similar results for the descending thoracic aorta.

**Figure 3:**
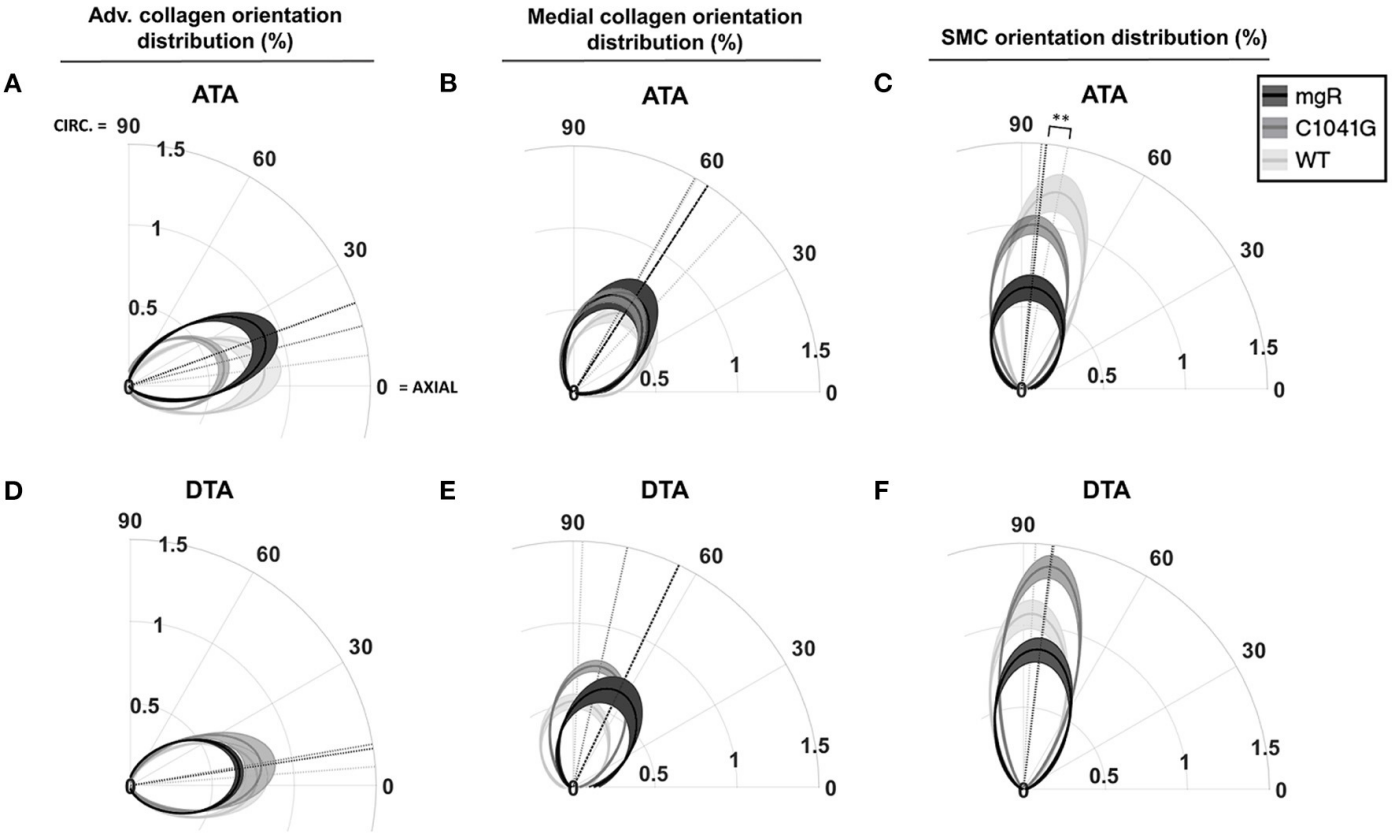
Quantification of in-plane orientation distributions of **(A,D)** adventitial and **(B,E)** medial collagen and similarly **(C,F)** medial smooth muscle cell nuclei under *in vivo* relevant diastolic conditions for the ascending (ATA, top row) and descending (DTA, bottom row) thoracic aorta for all three genotypes: WT (*n* = 5), C1041G (*n* = 7), and mgR (*n* = 6). In particular the polar plots indicate the mean primary orientations (radial dotted lines) and dispersion about the mean (radial narrowness or spread) of each genotype, each relative to the axial (horizontal) and circumferential (vertical) directions. The loaded collagen fibers were oriented primarily in the axial direction in the adventitia and in circumferential/diagonal directions in the media. Smooth muscle nuclei were oriented nearly circumferentially, with a gradual and dramatic increase in orientation dispersion by genotype (mgR > C1041G > WT) in the ATA but not in the DTA. Data are shown as average in-plane orientation distribution (solid line) and alignment SEM (shadow area), with **p < 0.01 for both primary orientation and alignment.

Given that the intra-lamellar and inter-lamellar organization of elastic fibers creates a sponge-like microstructure (12), we quantified these voids in terms of elastin porosity in three-dimensions (volume of medial elastic fiber voids/total enclosed elastic fiber volume). Figure 2 shows medial-to-adventitial ratios (panels 2A,E), layer-specific (intimal, medial, and adventitial) cell densities (2B,C,F), elastin porosity (2G), and two collagen characteristics (2D,H), all as a function of genotype for the ascending aorta. Importantly, there was a progressive increase in elastin porosity and decrease in smooth muscle cell density from WT to C1041G to mgR aortas. Only the mgR aortas, which exhibited greater dilatation, showed a significant reduction in endothelial cell density and increase in adventitial cell density relative to WT. The descending thoracic aorta exhibited similar histological features as the ascending aorta, though again with less severe changes (Supplementary Figure 2). Only endothelial cell density exhibited a slightly different trend in the two regions; it decreased gradually from WT to C1041G to mgR in the descending segment (Supplementary Figure 2B), while it was preserved in the ascending aorta of the C1041G but reduced in the mgR mice (Figure 2B). Both Marfan models tended to have straighter and thinner adventitial fiber bundles in the ascending (Figures 2D,H) and descending (Supplementary Figures 2D,H) segments, with altered orientations revealed by differences in both the preferred direction and degree of dispersion (Figures 3A,B,D,E). Along with their reduced density, the circumferential alignment of the smooth muscle cells decreased from WT to C1041G to mgR in the ascending but not the descending segment (Figures 3C,F), suggesting possible cell reorientation or morphological alteration with more severe disease.

**Figure 4:**
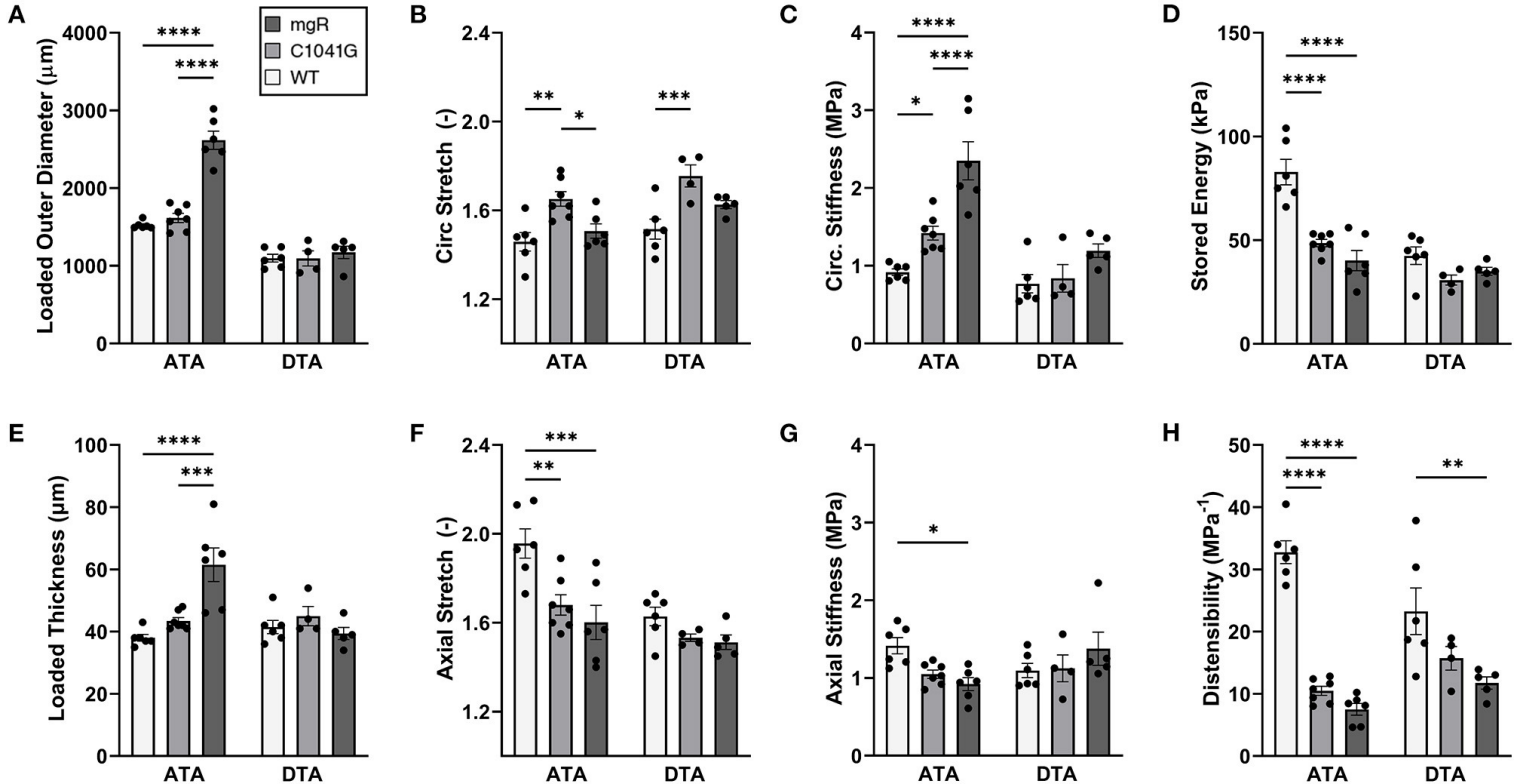
Multiple geometric and mechanical metrics derived from computer-controlled biaxial testing of passive ascending thoracic (ATA) and descending thoracic (DTA) aortas and calculated under diastolic conditions (80 mmHg and specimen-specific axial stretches) for all three genotypes: WT (*n* = 6), C1041G (*n* = 7), and mgR (*n* = 6). See Supplementary Figure 3 for similar calculations under systolic conditions, noting that the microstructural information in Figures 1–3 is for diastolic conditions. Note the similar trends for the two aortic segments, though more severe in the ascending aorta. In particular, there was a marked increase in **(A)** diameter and **(E)** wall thickness, a marked decrease in **(F)** axial stretch and **(D)** energy storage, and a marked increase in **(C)** circumferential material stiffness in the ascending aorta from WT to C1041G to mgR mice, that is, with increasing dilatation (recall Figure 1). See, too, results for axial **(B)** stretch and **(G)** stiffness plus **(H)** overall distensibility. Data are shown as mean ± SEM, with *p < 0.05, **p < 0.01, ***p < 0.001, and ****p < 0.0001. See Supplementary Figure 4 for similar results for wall stress.

*Mechanical changes associate with Marfan severity*. Eight associated geometric and mechanical metrics were computed for both the ascending and descending thoracic aorta under *ex vivo* simulated diastolic biaxial loading conditions for all three genotypes (Figures 4A–H); similar results under systolic loading are provided in Supplementary Material (Supplementary Figures 3A–H). Again, results trended similarly for the two thoracic aortic segments, but were less severe in the descending aorta. Focusing on the ascending aorta, luminal enlargement (Figure 4A), wall thickening (Figure 4E), and axial stretch reduction (Figure 4F) were each more severe in the mgR mice relative to C1041G and WT mice. Most importantly, however, there was a progressive increase in circumferential material stiffness (Figure 4C) and decrease in elastic energy storage (Figure 4D) from WT to C1041G to mgR mice, again less severe in the descending segment. Distensibility, an important clinical metric of structural stiffness also decreased significantly in the descending aorta and especially so in the ascending segment from WT to C1041G to mgR mice (Figure 4H). Similar results for biaxial wall stress as well as the associated non-linear stress-stretch behaviors are reported in Supplementary Figures 4A–F for both regions. See also Supplementary Table 1 for values (mean ± SEM) of each metric by genotype, many of which were calculated using best-fit values of the material parameters in the non linear constitutive relation (Supplementary Tables 2, 3; see also Methods).

**Figure 5:**
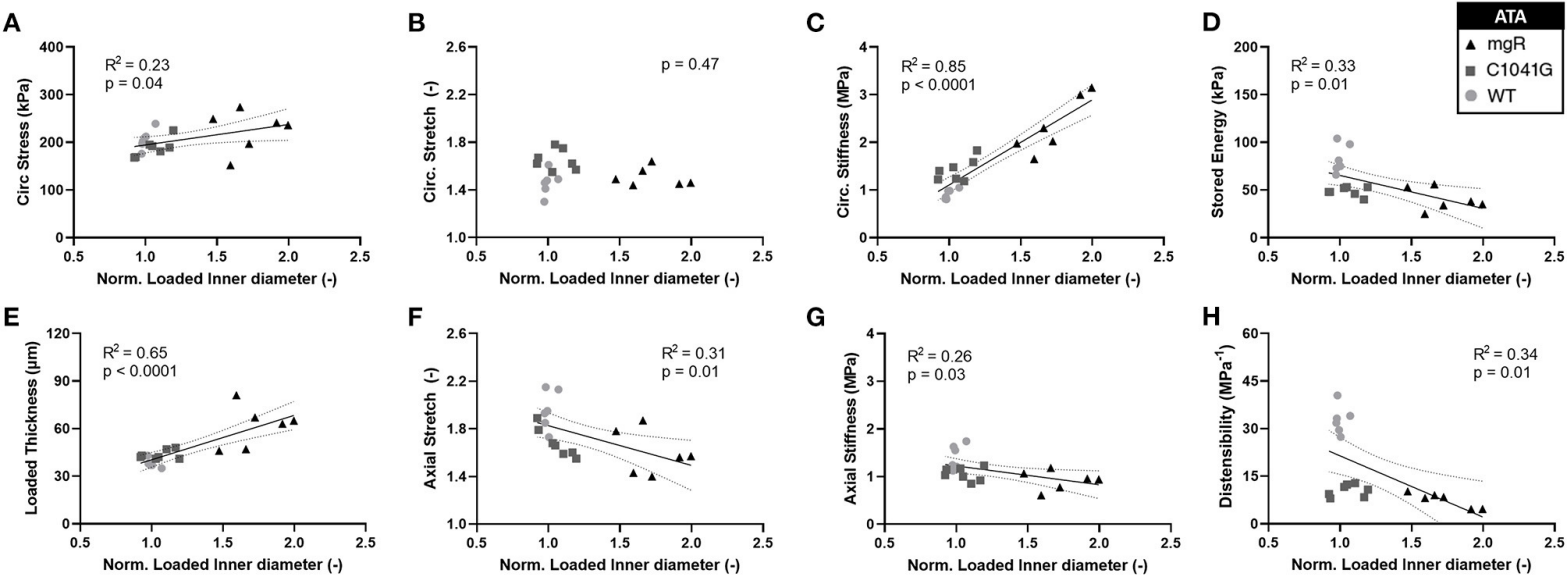
Plots of specimen-specific (*n* = 6 WT, *n* = 7 C1041G, *n* = 6 mgR) geometric and mechanical metrics vs. specimen-specific diameter for the ascending aorta, all calculated at *in vivo* relevant diastolic conditions of 80 mmHg and specimen-specific axial stretch. The data were fit by linear regression, with the best-fit line and 95% confidence intervals (solid and dotted lines, respectively) shown when the slope either was non-zero or showed a nearly significant trend. Note the particularly strong positive correlations for **(E)** increasing wall thickness and **(C)** increasing circumferential material stiffness. Note that all other results **(A, D, F–H)** except for **(B)** circumferential stretch were significant at *p* < 0.05. Similar results for the descending thoracic aorta are shown in Supplementary Figure 5.

Motivated by the qualitative observations of the microstructure as a function of the diameter as seen in Figure 1, each of these key mechanical metrics were then plotted as a function of aortic diameter normalized to mean wild-type diameter, independent of genotype (Figures 5A–H). The results show that changes in most mechanical metrics correlated well with increasing dilatation in the ascending aorta, particularly for increases in circumferential material stiffness (Figure 5C) and decreases in elastic energy storage (Figure 5D). These relationships are generally dominated by the large spread in diameter within the mgR group, whereas WT and C1041G are more similar in diameter. The descending thoracic aorta shows similar correlative results, again with the less severe phenotype evident by the lower correlations, if any (Supplementary Figures 5A–H).

**Figure 6:**
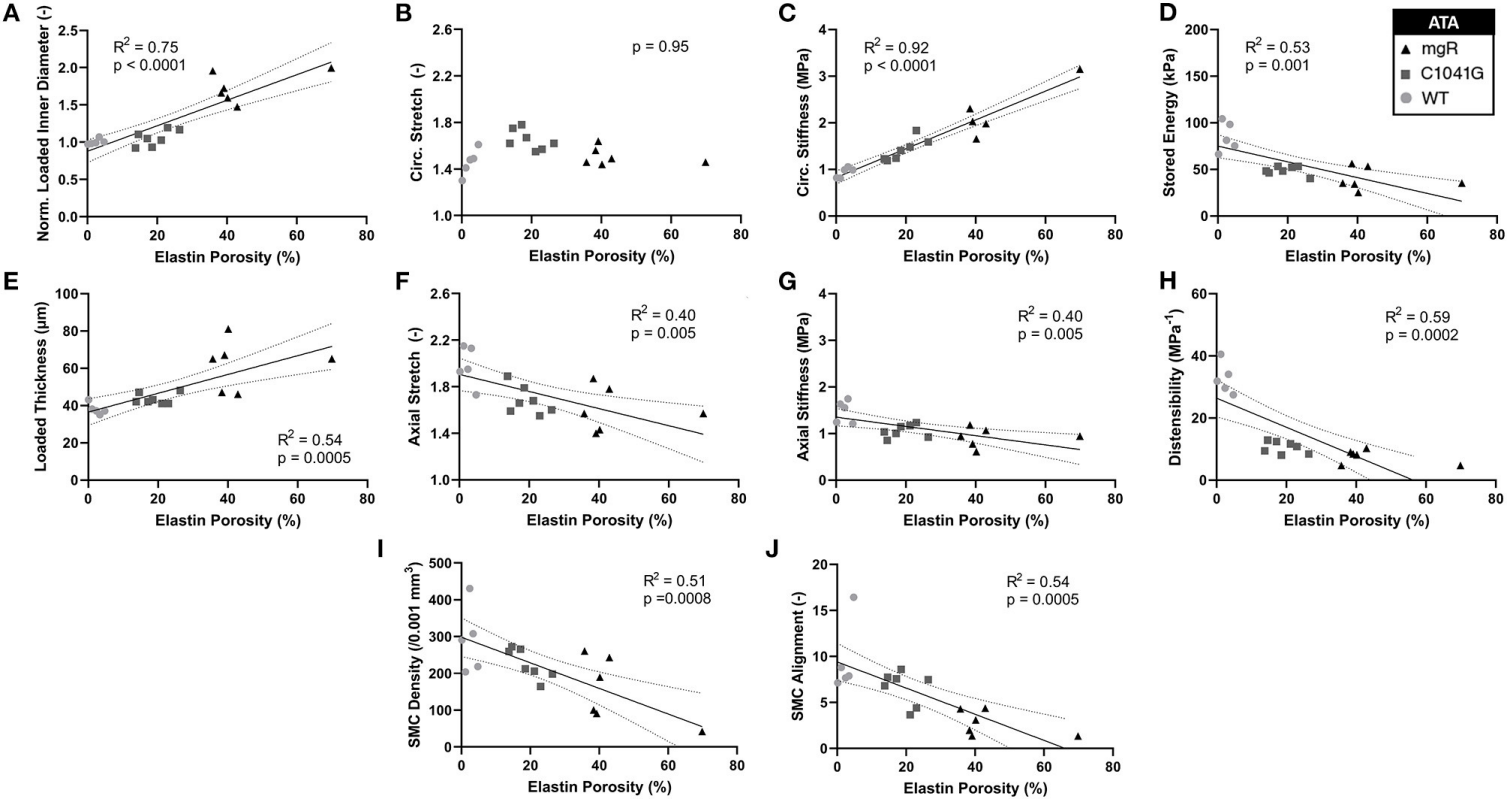
Plots of specimen-specific (*n* = 6 WT, *n* = 7 C1041G, *n* = 6 mgR) geometric and mechanical metrics for the ascending thoracic aorta (ATA) vs. specimen-specific elastin porosity, all calculated at *in vivo* relevant diastolic conditions of 80 mmHg and specimen-specific axial stretch. The data were fit by linear regression, with the best-fit line and 95% confidence intervals (solid and dotted lines, respectively) shown when the slope was either non-zero or showed a nearly significant trend. Recall that elastin porosity was shown in Figure 2G to increase dramatically by genotype (mgR > C1041G > WT). Notwithstanding the strong correlation between increasing diameter and elastin porosity **(A)**, note that most correlations here, **(C–J**, though not **B)** are even stronger than those in terms of diameter seen in Figure 5. Similar results for the descending thoracic aorta are shown in Supplementary Figure 6 while correlations for energy dissipation for both the ascending and descending thoracic aorta, which were also very strong, are shown in Supplementary Figure 8.

*Structure-function correlations highlight the detrimental role of increasing elastin porosity*. Given that mechanical properties result from the underlying microstructure, recall from Figure 2G the significant increase in elastin porosity from WT to C1041G to mgR ascending aortas. When plotting key specimen-specific geometric and mechanical metrics vs. elastin porosity, strong correlations emerged for most metrics for the ascending aorta (Figures 6A–H), but particularly so for increasing diameter (6A) and circumferential material stiffness (6C) as well as decreasing energy storage (6D). That is, these correlations are even stronger than those with normalized diameter, suggesting that the progressive medial deterioration reflected by the increasing elastin porosity going from WT to C1039G to mgR appears to dictate the increasing degree of mechanical dysfunction. Supplementary Figures 6A–H shows similar results for the descending thoracic aorta. In addition, decreases in endothelial cell density correlated strongly with increasing diameter, suggesting the proliferation did not keep pace with expanding luminal surface (Supplementary Figure 7). Finally, the progressive decrease in smooth muscle density and their altered alignment correlated with increasing elastin porosity (Figures 6I,J) and the dissipation of elastic energy revealed during cyclic pressure-loading similarly increased with both increasing normalized diameter (Supplementary Figures 8A,C) and increasing elastin porosity (Supplementary Figures 8B,D). These associations between morphological properties of the smooth muscle cells and the matrix constituents in which the cells are embedded motivated a further analysis of mechanical metrics vs. smooth muscle characteristics. Correlations between normalized diameter, circumferential stiffness, and stored elastic energy and smooth muscle alignment emerged for the ascending aortas (Supplementary Figures 9A–H). Although correlations also emerged when plotting mechanical metrics vs. other microstructural measures, including amount (cf. Figure 2) or orientation (cf. Figure 3), the complexity of the cellular (phenotype, orientation, and density) and collagen (type, orientation, undulation, and cross-linking) contributions to wall integrity renders it difficult to discern the most relevant or direct combinations of structural or functional characteristics.

## Discussion

Standard histology remains the mainstay of microstructural assessments of the aortic wall (13), and such studies consistently reveal fragmentation or loss of elastic lamellar structures within the medial layer in Marfan syndrome. Given the importance of fibrillin-1 to the structural integrity of the elastic fibers within these lamellar structures, it is not surprising that biomechanical properties of the aorta were also measured early on in multiple mouse studies. For example, Atkinson and colleagues reported increases in central pulse wave velocity, a measure of structural stiffening of the aortic wall, in *Fbn1*^*mgR*/*mgR*^ mice relative to WT. Using the Moens-Korteweg equation, they also inferred an increased circumferential material stiffness despite similar values of wall stress in this Marfan model (14). Van Breeman and colleagues used uniaxial ring tests to assess the mechanical properties of the aorta in the *Fbn1*^*C*1041*G*/+^ mouse. There are many limitations to uniaxial tests, but a lower failure stress in the aneurysmal aorta emerged in this mouse model relative to WT (15). More recently, Lessner and colleagues reported age- and sex-dependent differences in the ascending aorta in the *Fbn1*^*C*1041*G*/+^ mouse, including metrics based on multiphoton microscopy and uniaxial ring tests and a correlation between a so-called low-stretch stiffness and elastin breaks (16). Importantly, Sheppard and colleagues showed nicely in *Fbn1*^*mgR*/*mgR*^ mice that diminishing *in vivo* wall strain correlates strongly with breaks in the elastic lamellae, imaged via auto-fluorescence in histological sections (17). *In vivo* wall strain can thus be considered as a biomarker. In no case, however, has such correlation included biaxial mechanical metrics, which are particularly important for the ascending aorta since it experiences both distension and extension with every beat of the heart (18).

Notwithstanding the utility of standard histological assessments of fixed cross-sections of the aorta, modern imaging methods—microCT, multiphoton microscopy, and synchrotron imaging—provide many potential advantages, including the ability to image non-fixed vessels under *in vivo* relevant loading conditions. Vande Geest and colleagues reported both multiphoton-based findings and mechanical data for the descending thoracic aorta in *Fbn1*^*C*1041*G*/+^ mice. They found extensive disruptions to the elastic lamellar structures as well as decreased adventitial collagen waviness, which they attempted to relate to altered mechanical properties that, unfortunately, were analyzed incorrectly in terms of a slope of a non-conjugate Cauchy stress—Green strain plot (19). Van Breemen and colleagues reported similar disruptions to the elastic lamellar structures (20) while Tilton and colleagues showed that gaping holes exist in the elastic microstructure in the *Fbn1*^*mgR*/*mgR*^ aorta while the adventitial collagen displays a more dispersed appearance with straightened and thinner fibers (21). Also using multiphoton microscopy, Egea and colleagues showed further that the density and size of the lamellar fenestrations are greater in *Fbn1*^*C*1041*G*/+^ mice than in WT, particularly in the more proximal aorta (22). Using microCT they also showed intra-lamellar thickening in the presence of breaks in the elastic fibers, with holes within the lamellar structure increasing with age up to 9 months in *Fbn1*^*C*1041*G*/+^ mice (23). Our microstructural findings are consistent with these many reports. Notwithstanding the structural insights gleaned from these prior studies, again none attempted any detailed microstructural—mechanical correlations.

By contrast, we used multiple cyclic pressure-diameter and axial force-length testing protocols to subject each excised aorta to a broad range of physiologically relevant biaxial deformations and then computed and compared two key geometrical and eight mechanical metrics derived from a validated non linear constitutive relation: inner radius and wall thickness, circumferential and axial stretch, circumferential and axial wall stress, circumferential and axial material stiffness, elastically stored energy, and overall distensibility, all calculated at multiple physiological pressures and specimen-specific axial stretches. Of particular note, increased circumferential material stiffness has been shown to correlate well with aneurysmal propensity and presence (24) and decreased elastic energy storage has been found to track severity in elastopathies (25). Amongst the multiple metrics, we found increasing circumferential material stiffness to correlate most strongly with both increasing diameter and increasing elastin porosity with particularly strong correlations for decreasing energy storage emerging as well. Increased stiffness likely reflects both the loss of collagen undulation due to the loss of elastic fibers (26) that are pre-stretched during development and somatic growth and that continually seek to recoil in maturity (27) as well as adverse remodeling resulting from compromised cellular mechano-sensing or mechano-regulation of matrix (7). Noting that the energetically preferred extensibility (axial stretch) was reduced significantly in the ascending aorta of the Marfan mice, loss of energy storage similarly results from the stiffening of the wall that reduces biaxial deformations experienced by remaining competent elastic fibers as well as directly from the loss of elastic fibers.

Zhang and colleagues reported a detailed correlation, in the porcine thoracic aorta, between acute elastase-induced degradation of aortic elastin and results from biaxial mechanical testing. They found a transition between an increased distensibility and extensibility following mild elastin degradation and a marked decrease in distensibility and extensibility following complete degradation (28). These data, and others, reveal the importance not only of the contribution of competent elastic fibers to arterial properties, but also an intimate connection between elastic and collagen fibers, the latter of which stiffen the wall dramatically when straightened (26). Nevertheless, these acute studies could not account for the inevitable *in vivo* remodeling of collagen as elastic fibers degrade. Using a model of abdominal aortic aneurysms that combines acute elastase application with chronic beta-aminopropionitrile (BAPN) exposure, we found that progressive aortic enlargement following an initial insult to elastic fibers results in large part from rapid collagen turnover, noting that the new collagen need not have normal undulation due in part to the loss of pre-stretched elastic fibers (29). Our current findings did not quantify collagen turnover, but revealed marked re-organization within the media and especially in the adventitia, suggestive of increased turnover (Figure 3). We suggest, therefore, that the variable dilatation in these age-matched mice (Figure 1, left-to-right) may reflect a “pseudo-time” associated with progressive mural deterioration and luminal enlargement not balanced by smooth muscle and endothelial cell proliferation. Indeed, the correlation of smooth muscle loss with increasing elastin porosity is consistent with progressive anoikis, that is, apoptosis resulting from loss of cell-matrix connections. Regardless, these findings suggest that rates of matrix and cell turnover likely differ from mouse-to-mouse given that all vessels were excised at ~P60. It also appears that the elastic lamellar structure begins to deteriorate first within the inner media, then toward the outer media. Interestingly, Zhang and colleagues showed that the inner lamellar layers tend to be wavier than outer layers in the common carotid artery at sub-physiological distending pressures, resulting in the uniform stretching of the different layers under physiological pressures up to 120 mmHg (30), consistent with theoretical models (31). Preferential early micro-damage within the inner wall may thus depend in part on the more complex geometry and loading of the ascending aorta, which merits further consideration (32).

Although we emphasized changes in the ascending aorta, which are particularly important in Marfan syndrome, similar findings emerged for the descending thoracic aorta, though less dramatic. These findings are consistent with expectations based on prior comparisons of regional differences in elastic fiber deterioration (33) and wall mechanics (34) along the length of the *Fbn1*^*mgR*/*mgR*^ aorta. Of clinical importance, longitudinal studies in patients have revealed that the descending thoracic aorta presents with increasing aortopathy following surgical repair of the proximal aorta in Marfan patients (35). Although it is not known whether this increased risk arises because of changes in hemodynamic loading on the descending thoracic aorta following the surgical implantation of a stiff synthetic proximal graft or simply whether surgical extension of life-span allows a longer period for the descending segment to deteriorate, the importance of studying the descending aorta is nevertheless clear. Indeed, in some ways, these data may provide a comparative snapshot into early disease progression, particularly noting that most findings were qualitatively similar in the descending and ascending segments of the thoracic aorta, just quantitatively different, consistent with the hypothesis that continued deterioration would result in more dramatic changes not unlike that seen in the ascending aorta when comparing results for the milder C1041G and more severe mgR phenotypes.

Inasmuch as drug efficacy can depend on the age at initiation, as, for example, post-natal day P16 vs. P45 in mgR mice (36), there is a pressing need to quantify changes in microstructure and mechanics in these Marfan models as a function of maturation, noting that the aorta matures biomechanically by about P56 (37), which is one reason for our choice of P60. Most prior studies that included aging compared results in increasingly older (e.g., 3, 6, 9, and 12 months old) adult mice [e.g., (15, 16)], not developing mice. Another need is to correlate local rather than bulk changes in microstructure with local mechanical properties, which is now possible with advanced imaging methods (32) though not used herein. Indeed, with continuing advances in single cell RNA-sequencing and spatial-omics, there is also a need to co-localize gene expression, histological features, and material properties as a function of maturation and aging. Nonetheless, our correlations of bulk mechanical properties and microstructure relate better to that which is currently available clinically, namely, measures of distensibility based on changes in diameter over a cardiac cycle.

In conclusion, we emphasize that all segments of the aorta are exposed to cyclic biaxial loading, but especially the ascending aorta that distends and extends with each beat of the heart. This complex biaxial loading may contribute to the earlier onset of disease in the ascending segment, notwithstanding the potential role of smooth muscle embryonic lineage (38). If biaxial loading applied over extended periods is a key driver, then subsequent disease in other aortic segments should be expected when the ascending segment is repaired, that is, despite different embryonic cell origins, noting that smooth muscle cells of the carotid artery have a similar embryonic origin as many in the ascending aorta and yet the carotid typically does dilate in Marfan syndrome and it exhibits a mild biomechanical phenotype (39). Regardless, cyclic biaxial tests are necessary to quantify and compare the biomechanical properties of all aortic segments. The present data reveal strong correlations between compromised biaxial properties and progressive deterioration of elastic fibers within the enlarging Marfan aorta, with associated collagen remodeling insufficient in many cases to arrest continuing dilatation or expected rupture. Consistent with prior findings of multiphoton microscopy, the elastic fibers are not merely fragmented, they are grossly absent in local regions, thus giving the appearance of gaping holes quantified as well as an increased elastin porosity. Such holes may create intramural stress concentrations, which in turn could increase dissection or rupture risk (40), and via anoikis could contribute to medial cell drop out (41), which was demonstrated herein. This increased elastin porosity also correlated strongly with decreased elastic energy storage, that is aortic function, as well as increased circumferential material stiffness, likely due both to the loss of elastic-collagen fiber interactions (26) and compromised mechano-sensing and mechano-regulation of matrix (7). Increased stiffness can adversely affect the hemodynamics, including increased pulse wave velocities and thus increased central pulse pressures that further drive the aortic deterioration in an insidious positive feedback loop.

## Materials and Methods

### Animal Models

All animal procedures were approved by the Institutional Animal Care and Use Committee of Yale University. A total of 19 male mice, *n* = 6 wild-type control (C57BL/6J denoted WT, from Jax Mice), *n* = 7 *Fbn1*^*C*1041*G*/+^ (denoted C1041G, from Jax Mice), and *n* = 6 *Fbn1*^*mgR*/*mgR*^ (denoted mgR, derived from breeding pairs from Mt. Sinai Ichan School of Medicine) were euthanized by an intraperitoneal injection of Beuthanasia-D (150 mg/kg) at 8–9 weeks of age (~P60). This age was chosen to allow early disease development in C1041G mice (mild phenotype) without having an excessively low survival rate in mgR mice (severe phenotype), which at P60 approached 30%. Both the ascending thoracic aorta (ATA), from the aortic root to the brachiocephalic trunk, and the descending thoracic aorta (DTA), between the first and fourth pairs of intercostal branches, were excised carefully to preserve any branches for subsequent ligation. Loose peri-adventitial tissue was removed by blunt dissection and the samples were placed within a custom, computer-controlled device for mechanical testing and microstructural assessment.

### Microstructural Assessment

Multiphoton microscopy was performed while the specimen was immersed in a Hanks buffered saline solution (HBSS) at room temperature to minimize smooth muscle contraction, then secured on custom glass cannulae, and held at its specimen-specific axial stretch but a common intraluminal pressure of 80 mmHg. The microscope (LaVision Biotec TriMScope) was powered by a Titanium-Sapphire laser (Chameleon Vision II, Coherent) tuned at 840 nm and equipped with a 20X water immersion objective lens (N.A. 0.95). Signals from three major components of the aortic wall were acquired simultaneously: the second harmonic generation (SHG) signal arising from fibrillar collagens (390–425 nm), two-photon excited fluorescence (TPF) from elastin (500–550 nm), and a fluorescent signal of cell nuclei (above 550 nm). Three-dimensional (3D) images were acquired with an in-plane (axial-circumferential) field of view of 500 μm × 500 μm at a consistent anatomical location. The acquired volume always corresponded to the center of the outer curvature in the case of the ATA and to the center of the ventral region between the second and third pair of intercostal arteries in the case of the DTA. Numerical imaging resolution was 0.48 μm/pixel, while the out-of-plane (radial axis) step size was 1 μm/pixel. Each image was acquired and pre-processed as previously described (42).

Layer-specific thickness was quantified by measuring the mean intensity profiles along the radial direction for 25 subunits of volume uniformly distributed across the axial-circumferential plane of the 3D images. The internal and external limits of the wall were identified as the limit for cell nuclei and limit for adventitial collagen, respectively, by automatically selecting the radial positions beyond which the area under the two tails of the normalized intensity profile corresponded to defined thresholds. The interface between adventitia and media was defined as the radial position of the maximum derivative of the intensity profile of the nucleus signal between the radial position of the maximum of the collagen intensity profile and the inner limit of the wall.

New here, within the radial range of the media, elastin porosity was quantified as the ratio between the volume identified as voids and the volume occupied by elastic fibers. For this purpose, the previously measured mean intensity profile of the elastin signal was used to radially exclude the interlamellar spaces by removing from the quantification the radial planes identified as local minima and their ±1 μm neighbor radial planes. An adaptive 3D filter (43) was applied to the remaining volume to reduce possible inhomogeneities due to local waviness of the elastic lamellae, the image was binarized using the automatic local Phansalkar thresholding method (44), and the volume of the identified voids was quantified as a black-to-white ratio.

Collagen fiber bundle characterization focused on in-plane parameters: straightness, bundle width, primary orientation, and alignment. Straightness was computed as the ratio of end-to-end to total fiber length for 25 fibers, automatically selected at each fixed depth for each image; bundle width (transversal section in the axial-circumferential plane) was measured directly; the in-plane primary orientation and alignment were estimated via a distribution of orientations for each circumferential-axial section using a 2D structure tensor analysis, OrientationJ plug-in for ImageJ. This distribution was averaged along the radial direction and normalized. Distributions were parameterized with a Von Mises circular probability density function *F*(θ∣μ, κ) = exp(κcos(θ−μ))/2π*I*_0_(κ), where μ is the primary orientation, *I*_0_ a modified Bessel function, and κ the alignment parameter, a measure of concentration that quantifies fiber alignment at the tissue level. The resulting functions are displayed in polar coordinates, where the radial dimension indicates the normalized orientation distribution.

Three cell groups were considered based on characteristic shapes of their nuclei and radial location: endothelial (monolayer on intima), smooth muscle (within the media), and cells within the adventitia. Medial and adventitial cell density was calculated as the number of cells per unit volume by counting the number of nuclei within defined sub-volumes of the 3D image and normalizing by the appropriate sample-specific thickness. A semi-automatic algorithm selected multiple subunits of volume (100 μm × 100 μm x local layer-specific thickness, *n* = 25) for each 3D image, with conversion to binary form by filtering and thresholding. Luminal cell density was computed as number of cells per surface area and normalized by 0.01 mm^2^; medial and adventitial cell numbers were normalized by 0.001 mm^3^, each to allow consistent comparisons. Additional details can be found elsewhere (42).

We show together in Figure 1 and Supplementary Figure 1A the results from all three channels, namely the collagen fibers from SHG (red), the elastic fibers from TPF (gray), and the cell nuclei (blue), though overlaying the cell nuclei tends to mask in part the detail available for the matrix (cf. Supplementary Figure 1B) that was otherwise quantified well as presented in Figures 2, 3, and Supplementary Figure 2.

### Mechanical Testing

Arteries are subjected to complex multiaxial loading *in vivo*, with the primary directions of loading being circumferential and axial. Cyclic pressure-diameter and axial force-length tests on cylindrical segments of excised arteries are thus preferred for quantifying *in vivo* relevant mechanical properties (45). All passive biaxial mechanical testing and data analyses were performed using validated methods (24, 46). Briefly, excised aortic segments were cannulated and immersed in a HBSS solution at room temperature (to minimize smooth muscle contraction), preconditioned via cyclic pressurization while held at their energetically preferred (*in vivo*) axial stretch, $\lambda_{z}^{iv}$, then subjected to a sequence of seven biaxial protocols: cyclic pressurization tests from 10 to 140 mmHg at three fixed axial stretches ($\lambda_{z}^{iv}$ and ± 5% of this value) and cyclic axial loading tests from 0 to *f*_max_ at four fixed luminal pressures (10, 60, 100, 140 mmHg), where *f*_max_ (in mN) was the specimen-specific value reached at a pressure of 140 mmHg and 1.05 $\lambda_{z}^{iv}$. The unloading portions of the last cycle of all seven pressure-diameter and axial force-length tests were fit simultaneously with a validated four-fiber family constitutive model using a Marquardt-Levenberg non linear regression of the data. Using data during unloading reveals the elastic energy stored during deformation that would be available to work on the distending fluid, noting that the primary function of conduit arteries such as the aorta is to store energy elastically during systolic distension and to use this energy during diastole to work on the blood and augment flow. By contrast, energy dissipation is computed via the difference in the loading and unloading curves. The four-fiber family constitutive relation is written in terms of a scalar pseudoelastic stored energy function *W*, namely


$$\begin{array}{l} W\left(\text{C},{\text{M}}^{i}\right)=\frac{c}{2}\left(I_{C}-3\right)+\overset{4}{\underset{i=1}{\sum }}\frac{c_{1}^{i}}{{4c}_{2}^{i}}\left\{\exp\left[c_{2}^{i}{\left(IV_{C}^{i}-1\right)}^{2}\right]-1\right\}, \end{array}$$

where the eight model parameters to be determined via regression are *c* (kPa), $c_{1}^{i}$ (kPa), and $c_{2}^{i}$ (-), with *i* = 1, 2, 3, 4 denoting the four predominant fiber family directions. *I*_*C*_ = *tr*(**C**) and $IV_{C}^{i}={\text{M}}^{i}\cdot \text{C}{\text{M}}^{i}$ are coordinate invariant measures of the finite deformation, with the right Cauchy-Green tensor **C** = **F**^*T*^**F** computed from the experimentally measured deformation gradient tensor **F** = *diag*[λ_*r*_, λ_θ_, λ_*z*_], with det**F** = 1 because of assumed incompressibility. The direction of the *i*^*th*^ family of fibers was prescribed by ${\text{M}}^{i}=\left[0,\;\sin\alpha_{0}^{i},\;\cos\alpha_{0}^{i}\right]$, with $\alpha_{0}^{i}$ denoting a fiber angle relative to the axial direction in the traction-free reference configuration. Because of the yet unknown effects of cross-links amongst the multiple families of fibers, the four predominant families were: axial ($\alpha_{0}^{1}=0$), circumferential ($\alpha_{0}^{2}=\frac{\pi }{2}$), and symmetric diagonal ($\alpha_{0}^{3,4}={\pm \alpha }_{0}$). Values of mean biaxial wall stress and material stiffness were computed from the stored energy function and calculated at individually measured values of blood pressure or a common pressure.

### Statistics

All grouped mechanical and microstructural metrics are presented as means ± SEM. Statistical analyses among experimental groups were performed using one-way analysis of variance (ANOVA), followed by Tukey's *post-doc* tests in case of statistical significance. A *p*-value below 0.05 was considered statistically significant. Correlation between different metrics was carried out computing the Pearson correlation coefficient, the square root of the coefficient of determination *R*^2^. Graphs were created using Graphpad Prism 9.0.

## Data Availability Statement

The original contributions presented in the study are included in the article/Supplementary Material, further inquiries can be directed to the corresponding author/s.

## Ethics Statement

The animal study was reviewed and approved by Institutional Animal Care and Use Committee of Yale University.

## Author Contributions

CC and JH conceptualized the study. CC, MC, DW, and MR-R contributed to animal care and specimen preparation, CC, DW, and MR-R performed biomechanical testing. CC performed microscopy acquisition and analysis. CC, MC, and MR-R contributed to the conception and implementation of the microstructural analysis. CC and JH prepared the figures and wrote the manuscript. All authors contributed to the interpretation of the overall results, discussed the results, and contributed to the final manuscript.

## Funding

This work was supported, in part, by a program project grant from the US National Institutes of Health (P01 HL134605, D.B. Rifkin PI).

## Conflict of Interest

The authors declare that the research was conducted in the absence of any commercial or financial relationships that could be construed as a potential conflict of interest.

## Publisher's Note

All claims expressed in this article are solely those of the authors and do not necessarily represent those of their affiliated organizations, or those of the publisher, the editors and the reviewers. Any product that may be evaluated in this article, or claim that may be made by its manufacturer, is not guaranteed or endorsed by the publisher.

## References

[1] Ramirez F Dietz HC. Fibrillin-rich microfibrils: structural determinants of morphogenetic and homeostatic events. J Cell Physiol. (2007) 213:326–30. 10.1002/jcp.21189 17708531

[2] Pereira L Lee SY Gayraud B Andrikopoulos K Shapiro SD Bunton T. Pathogenetic sequence for aneurysm revealed in mice underexpressing fibrillin-1. PNAS. (1999) 96:3819–23. 10.1073/pnas.96.7.3819 10097121 PMC22378

[3] Davis EC. Stability of elastin in the developing mouse aorta: a quantitative radioautographic study. Histochemistry. (1993) 100:17–26. 10.1007/BF00268874 8226106

[4] Yanagisawa H Wagenseil J. Elastic fibers and biomechanics of the aorta: insights from mouse studies. Matrix Biol. (2020) 85–86:160–72. 10.1016/j.matbio.2019.03.001 30880160 PMC6745291

[5] Chaudhry SS Cain SA Morgan A Dallas SL Shuttleworth CA Kielty CM. Fibrillin-1 regulates the bioavailability of TGFbeta1. J Cell Biol. (2007) 176:355–67. 10.1083/jcb.200608167 17242066 PMC2063961

[6] Doyle JJ Gerber EE Dietz HC. Matrix-dependent perturbation of TGFβ signaling and disease. FEBS Lett. (2012) 586:2003–15. 10.1016/j.febslet.2012.05.027 22641039 PMC3426037

[7] Humphrey JD Schwartz MA Tellides G Milewicz DM. Role of mechanotransduction in vascular biology. Circ Res. (2015) 116:1448–61. 10.1161/CIRCRESAHA.114.304936 25858068 PMC4420625

[8] Carta L Wagenseil JE Knutsen RH Mariko B Faury G Davis EC. Discrete contributions of elastic fiber components to arterial development and mechanical compliance. Arterioscl Thromb Vasc Biol. (2009) 29:2083–9. 10.1161/ATVBAHA.109.193227 19850904 PMC2797476

[9] Mariko B Pezet M Escoubet B Bouillot S Andrieu J.-P.. Fibrillin-1 genetic deficiency leads to pathological ageing of arteries in mice. J Pathol. (2011) 224:33–44. 10.1002/path.2840 21432852 PMC3075583

[10] Milewicz DM Braverman AC De Backer J Morris SA Boileau C Maumenee IH. Marfan syndrome. Nat Rev Dis Primers. (2021) 7:64. 10.1038/s41572-021-00298-7 34475413 PMC9261969

[11] Judge DP Biery NJ Keene DR Geubtner J Myers L Huso DL. Evidence for a critical contribution of haploinsufficiency in the complex pathogenesis of marfan syndrome. J Clin Invest. (2004) 114:172–81. 10.1172/JCI200420641 15254584 PMC449744

[12] Nakashima Y Sueishi K. Alteration of elastic architecture in the lathyritic rat aorta implies the pathogenesis of aortic dissecting aneurysm. Am J Pathol. (1992) 140:959–69. 1562054 PMC1886377

[13] Halushka MK Angelini A Bartoloni G Basso C Batoroeva L Bruneval P. Consensus statement on surgical pathology of the aorta from the society for cardiovascular pathology and the association for european cardiovascular pathology: II. Noninflammatory degenerative diseases—nomenclature and diagnostic criteria. Cardiovasc Pathol. (2016) 25:247–57. 10.1016/j.carpath.2016.03.002 27031798

[14] Marque V Kieffer P Gayraud B Lartaud-Idjouadiene I Ramirez F Atkinson J. Aortic wall mechanics and composition in a transgenic mouse model of marfan syndrome. Arterioscl Thromb Vasc Biol. (2001) 21:1184–9. 10.1161/hq0701.092136 11451749

[15] Chung AWY Au Yeung K Sandor GGS Judge DP Dietz HC van Breemen C. Loss of elastic fiber integrity and reduction of vascular smooth muscle contraction resulting from the upregulated activities of matrix metalloproteinase-2 and−9 in the thoracic aortic aneurysm in marfan syndrome. Circ Res. (2007) 101:512–22. 10.1161/CIRCRESAHA.107.157776 17641224

[16] Gharraee N Sun Y Swisher JA Lessner SM. Age and sex dependency of thoracic aortopathy in a mouse model of marfan syndrome. Am J Physiol Heart Circ Physiol. (2021). 10.1152/ajpheart.00255.2021. [Epub ahead of print]. 34714692 PMC8698500

[17] Chen JZ Sawada H Moorleghen JJ Weiland M Daugherty A Sheppard MB. Aortic strain correlates with elastin fragmentation in fibrillin-1 hypomorphic mice. Circ Rep. (2019) 1:199–205. 10.1253/circrep.CR-18-0012 31123721 PMC6528667

[18] Ferruzzi J Achille PD Tellides G Humphrey JD. Combining *in vivo* and *in vitro* biomechanical data reveals key roles of perivascular tethering in central artery function. PLoS ONE. (2018) 13:e0201379. 10.1371/journal.pone.0201379 30192758 PMC6128471

[19] Haskett D Doyle JJ Gard C Chen H Ball C Estabrook MA. Altered tissue behavior of a non-aneurysmal descending thoracic aorta in the mouse model of marfan syndrome. Cell Tissue Res. (2012) 347:267–77. 10.1007/s00441-011-1270-y 22105919

[20] Cui JZ Tehrani AY Jett KA Bernatchez P van Breemen C Esfandiarei M. Quantification of aortic and cutaneous elastin and collagen morphology in marfan syndrome by multiphoton microscopy. J Struct Biol. (2014) 187:242–53. 10.1016/j.jsb.2014.07.003 25086405

[21] Ju X Ijaz T Sun H Lejeune W Vargas G Shilagard T. IL-6 regulates extracellular matrix remodeling associated with aortic dilation in a fibrillin-1 hypomorphic mgR/mgR mouse model of severe marfan syndrome. J Am Heart Assoc. (2014) 3:e000476. 10.1161/JAHA.113.000476 24449804 PMC3959679

[22] López-Guimet J Andilla J Loza-Alvarez P Egea G. High-resolution morphological approach to analyse elastic laminae injuries of the ascending aorta in a murine model of marfan syndrome. Sci Rep. (2017) 7:1505. 10.1038/s41598-017-01620-8 28473723 PMC5431420

[23] López-Guimet J Peña-Pérez L Bradley RS García-Canadilla P Disney C Geng H. MicroCT imaging reveals differential 3D micro-scale remodelling of the murine aorta in ageing and marfan syndrome. Theranostics. (2018) 8:6038–52. 10.7150/thno.26598 30613281 PMC6299435

[24] Bellini C Bersi MR Caulk AW Ferruzzi J Milewicz DM Ramirez F. Comparison of 10 murine models reveals a distinct biomechanical phenotype in thoracic aortic aneurysms. J Royal Soc Interface. (2017) 14:20161036. 10.1098/rsif.2016.1036 28490606 PMC5454287

[25] Humphrey JD Tellides G. Central artery stiffness and thoracic aortopathy. Am J Physiol Heart Circ Physiol. (2019) 316:H169–82. 10.1152/ajpheart.00205.2018 30412443 PMC6880196

[26] Ferruzzi J Collins MJ Yeh AT Humphrey JD. Mechanical assessment of elastin integrity in fibrillin-1-deficient carotid arteries: implications for marfan syndrome. Cardiovasc Res. (2011) 92:287–95. 10.1093/cvr/cvr195 21730037 PMC3193833

[27] Cardamone L Valentín A Eberth JF Humphrey JD. Origin of axial prestretch and residual stress in arteries. Bio Model Mechanobiol. (2009) 8:431. 10.1007/s10237-008-0146-x 19123012 PMC2891240

[28] Chow M-J Mondonedo JR Johnson VM Zhang Y. Progressive structural and biomechanical changes in elastin degraded aorta. Biomech Model Mechanobiol. (2013) 12:361–372. 10.1007/s10237-012-0404-9 22623109 PMC3568224

[29] Weiss D Latorre M Rego BV Cavinato C Tanski BJ Berman AG. Biomechanical consequences of compromised elastic fiber integrity and matrix cross-linking on abdominal aortic aneurysmal enlargement. Acta Bio. (2021) 134:422–34. 10.1016/j.actbio.2021.07.059 34332103 PMC8542633

[30] Yu X Turcotte R Seta F Zhang Y. Micromechanics of elastic lamellae: unravelling the role of structural inhomogeneity in multi-scale arterial mechanics. J R Soc Interface. (2018) 15:20180492. 10.1098/rsif.2018.0492 30333250 PMC6228495

[31] Bellini C Ferruzzi J Roccabianca S Di Martino ES Humphrey JD. A microstructurally motivated model of arterial wall mechanics with mechanobiological implications. Ann Biomed Eng. (2014) 42:488–502. 10.1007/s10439-013-0928-x 24197802 PMC3943530

[32] Bersi MR Bellini C Humphrey JD Avril S. Local variations in material and structural properties characterize murine thoracic aortic aneurysm mechanics. Biomech Model Mechanobiol. (2019) 18:203–18. 10.1007/s10237-018-1077-9 30251206 PMC6367054

[33] Schwill S Seppelt P Grünhagen J Ott C.-E. Jugold M. The fibrillin-1 hypomorphic mgR/mgR murine model of marfan syndrome shows severe elastolysis in all segments of the aorta. J Vasc Surg. (2013) 57:1628–36 1636.e1–3. 10.1016/j.jvs.2012.10.007 23294503

[34] Bellini C Korneva A Zilberberg L Ramirez F Rifkin DB Humphrey JD. Differential ascending and descending aortic mechanics parallel aneurysmal propensity in a mouse model of marfan syndrome. J Biomech. (2016) 49:2383–9. 10.1016/j.jbiomech.2015.11.059 26755343 PMC4917480

[35] Palumbo MC Rong LQ Kim J Navid P Sultana R Butcher J. Prosthetic aortic graft replacement of the ascending thoracic aorta alters biomechanics of the native descending aorta as assessed by transthoracic echocardiography. PLoS ONE. (2020) 15:e0230208. 10.1371/journal.pone.0230208 32163486 PMC7067394

[36] Cook JR Clayton NP Carta L Galatioto J Chiu E Smaldone S. Dimorphic effects of transforming growth factor-β signaling during aortic aneurysm progression in mice suggest a combinatorial therapy for marfan syndrome. Arterioscl Thromb Vasc Biol. (2015) 35:911–7. 10.1161/ATVBAHA.114.305150 25614286 PMC4376614

[37] Murtada S.-I., Kawamura Y, Li G, Schwartz MA, Tellides G, et al. Developmental origins of mechanical homeostasis in the aorta. Dev Dynamics. (2021) 250:629–39. 10.1002/dvdy.283 33341996 PMC8089041

[38] Sawada H Rateri DL Moorleghen JJ Majesky MW Daugherty A. Smooth muscle cells derived from second heart field and cardiac neural crest reside in spatially distinct domains in the media of the ascending aorta—brief report. Arterioscl Thromb Vasc Biol. (2017) 37:1722–6. 10.1161/ATVBAHA.117.309599 28663257 PMC5570666

[39] Eberth JF Taucer AI Wilson E Humphrey JD. Mechanics of carotid arteries in a mouse model of marfan syndrome. Ann Biomed Eng. (2009) 37:1093–104. 10.1007/s10439-009-9686-1 19350391 PMC2753508

[40] Roccabianca S Figueroa CA Tellides G Humphrey JD. Quantification of regional differences in aortic stiffness in the aging human. J Mech Behav Biomed Mater. (2014) 29:618–34. 10.1016/j.jmbbm.2013.01.026 23499251 PMC3842391

[41] Michel J-B Jondeau G Milewicz DM. From genetics to response to injury: vascular smooth muscle cells in aneurysms and dissections of the ascending aorta. Cardiovasc Res. (2018) 114:578–89. 10.1093/cvr/cvy006 29360940 PMC6658716

[42] Cavinato C Murtada S-I Rojas A Humphrey JD. Evolving structure-function relations during aortic maturation and aging revealed by multiphoton microscopy. Mechan Ageing Dev. (2021) 196:111471. 10.1016/j.mad.2021.111471 33741396 PMC8154707

[43] Ollion J Cochennec J Loll F Escud é C Boudier T. TANGO: a generic tool for high-throughput 3D image analysis for studying nuclear organization. Bioinformatics. (2013) 29:1840–1. 10.1093/bioinformatics/btt276 23681123 PMC3702251

[44] Neerad P Sumit M Ashish S Madhuri J. Adaptive local thresholding for detection of nuclei in diversity stained cytology images. in 2011 International Conference on Communications and Signal Processing. (2011). 218–20.

[45] Humphrey JD. Cardiovascular Solid Mechanics: Cells, Tissues, and Organs. New York, NY: Springer-Verlag (2002).

[46] Ferruzzi J Bersi MR Uman S Yanagisawa H Humphrey JD. Decreased elastic energy storage, not increased material stiffness, characterizes central artery dysfunction in fibulin-5 deficiency independent of sex. J Biomech Eng. (2015) 137:0310071. 10.1115/1.4029431 25532020 PMC4321117

