# Supplementary material for: Progressive Microstructural Deterioration Dictates Evolving Biomechanical Dysfunction in the Marfan Aorta
Source: Front Cardiovasc Med. 2021 Dec 16;8:800730. doi: 10.3389/fcvm.2021.800730 (PMC8716484; doi:10.3389/fcvm.2021.800730)
Supplement: Supplementary file 1 [file Data_Sheet_1.pdf]

Supplementary Material

Progressive Microstructural Deterioration Dictates Evolving Biomechanical Dysfunction in the Marfan Aorta

Cristina Cavinato[1], Minghao Chen[2], Dar Weiss[1], Maria Jesús Ruiz-Rodríguez[3],

Martin A. Schwartz[1,2], Jay D. Humphrey[1,4*]

[1]Department of Biomedical Engineering, Yale University, New Haven, CT, USA

[2]Cardiovascular Research Center and Department of Internal Medicine (Cardiology), Yale School of Medicine, New Haven, CT, USA

[3]Centro Nacional de Investigaciones Cardiovasculares (CNIC), Madrid, Spain

[4]Vascular Biology and Therapeutics Program, Yale School of Medicine, New Haven, CT, USA

*** Correspondence:** jay.humphrey@yale.edu

1.1 Supplementary Figures

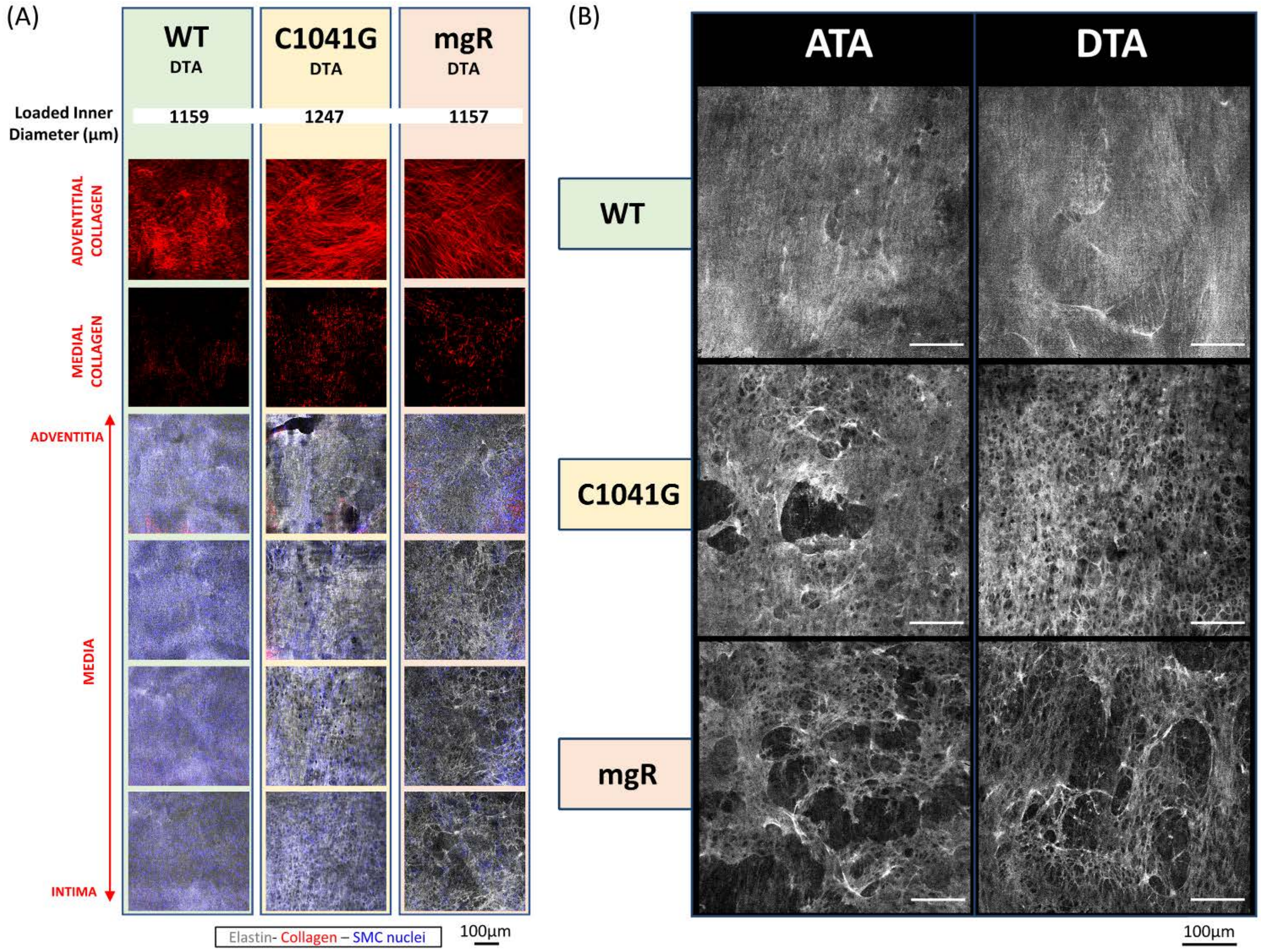

Supplementary Figure 1. (A) Mosaic of representative images from multiphoton microscopy of the descending thoracic aorta (DTA): one wild-type (WT), *Fbn1*$^{C1041G/+}$ (C1041G), and *Fbn1*$^{mgR/mgR}$ (mgR) mouse each, from left-to-right. Note that the images were collected ex vivo under in vivo relevant diastolic conditions, namely, at an 80-mmHg distending pressure and specimen-specific in vivo value of axial stretch. Shown from top-to-bottom, adventitial then medial fibrillar collagen (red, via second harmonic generation) and four equally spaced medial elastin volumes (gray, two-photon fluorescence images), the latter with superimposed cell nuclei (blue) which mask, in part, the underlying detail on the elastin but are critical to compare given the marked loss of cells in the Marfan aortas in addition to the increased porosity of the elastic structures. Compare with Figure 1 in the main text, which shows similar findings for the ascending thoracic aorta (ATA). (B) Higher magnification of elastin images, sectioned at half thickness of the medial layer for one representative sample of each group and region (ATA on the left, DTA on the right) of the thoracic aorta, shows more clearly the general deterioration of the elastin architecture from the WT to the C1041G to the mgR mice (top to bottom).

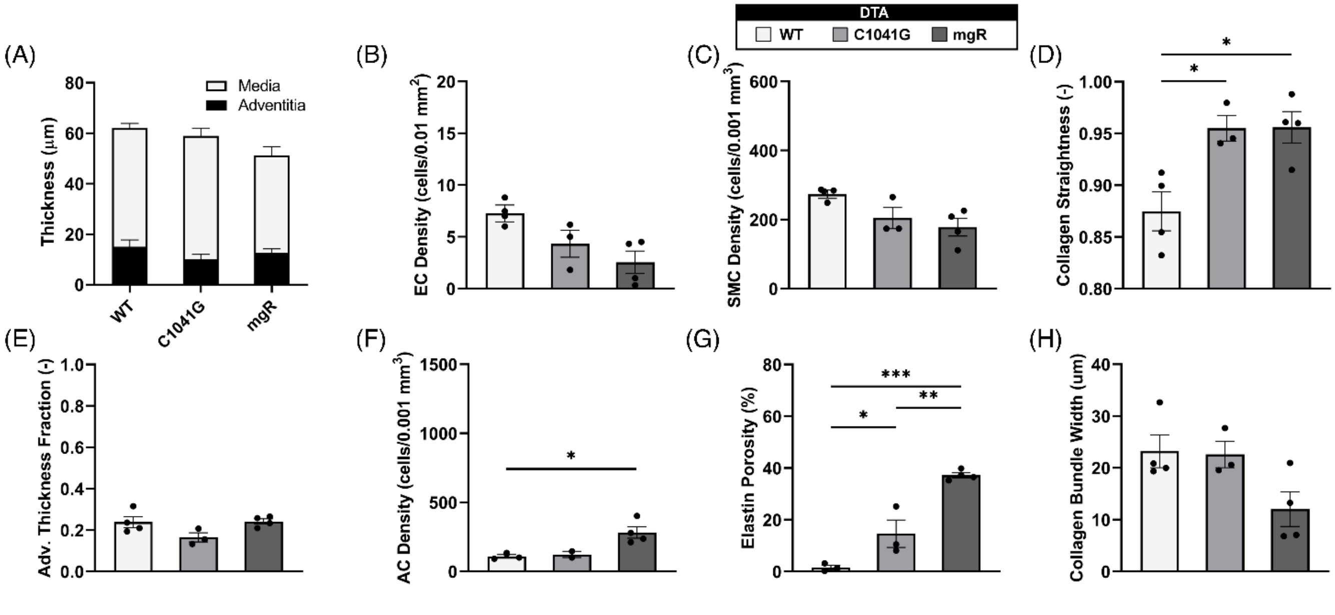

Supplementary Figure 2. Multiple microstructural metrics for the descending thoracic aorta (DTA) plotted as a function of genotype: WT (*n*=4), C1041G (*n*=3), and mgR (*n*=4). Included are (A) medial and adventitial thicknesses, and (E) their ratio, plus (B, C, F) layer-specific cell densities based on cell nuclei and associated areas or volumes as well as (G) elastin porosity and (D, H) two measures of collagen fiber structure (straightness and fiber bundle width). Data are shown as mean ± SEM, with * $p < 0.05$.

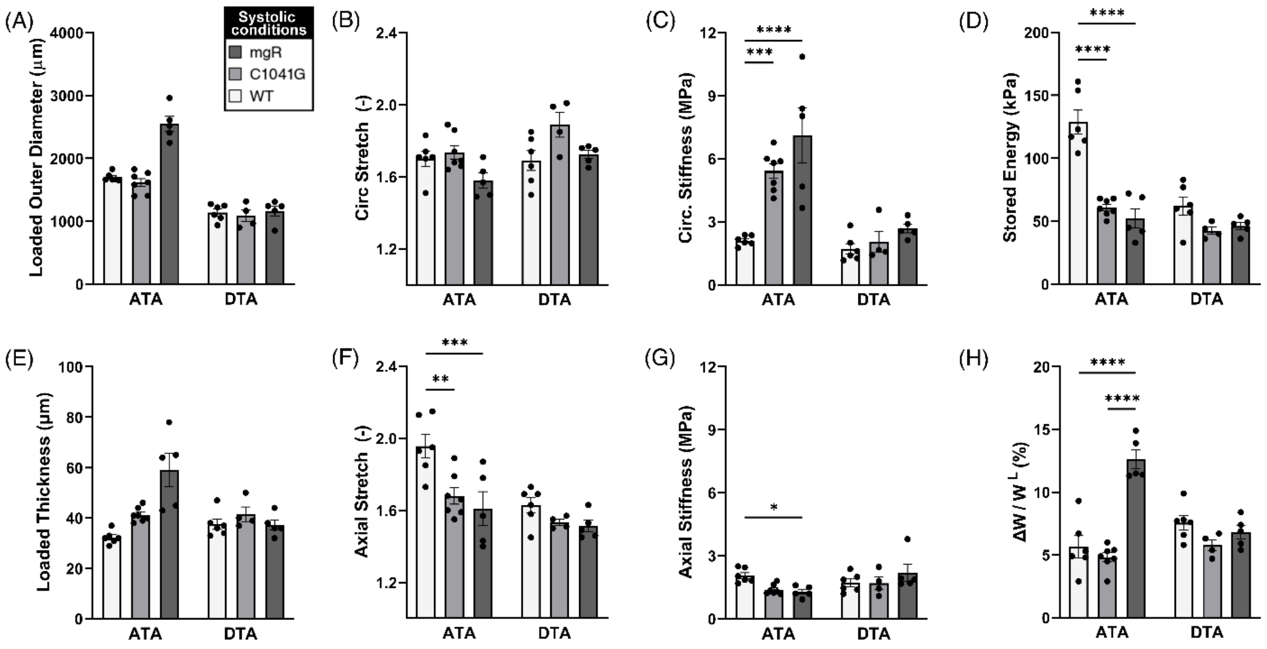

Supplementary Figure 3. Multiple geometric and mechanical metrics derived from computer-controlled biaxial testing of passive ascending (ATA) and descending (DTA) thoracic aortas calculated under systolic conditions (120 mmHg and specimen-specific axial stretches) for all three genotypes: WT (*n*=6 for the ATA and 6 for the DTA), C1041G (*n*=7 and 4), and mgR (*n*=5 and 5). Note the similar trends for the two aortic segments, though more severe in the ascending aorta. In particular, there was a marked increase in (A) diameter and (E) wall thickness, a marked decrease in (F) axial stretch and (D) energy storage, and a marked increase in (C) circumferential material stiffness and (H) energy dissipation in the ascending aorta from WT to C1041G to mgR mice, that is, with increasing dilatation (recall Figure 1). Less clear trends were observed for (B) circumferential stretch, (G) axial stiffness, and the mechanical data in the descending thoracic aorta. Data are shown as mean ± SEM, with * $p < 0.05$, ** $p < 0.01$, *** $p < 0.001$ and **** $p < 0.0001$.

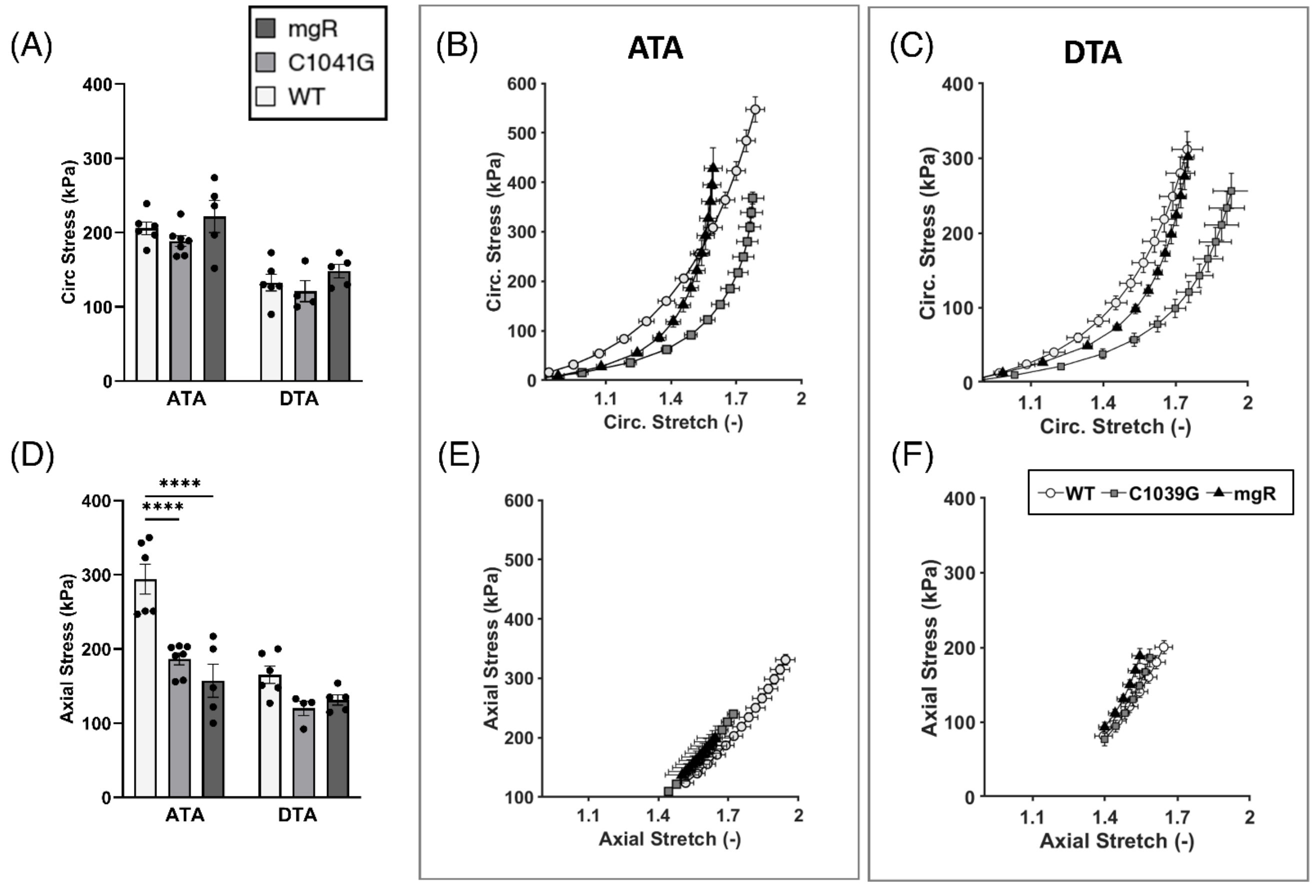

Supplementary Figure 4. Biaxial wall stress (A, D) derived from computer-controlled biaxial testing of passive ascending and descending thoracic aortas and calculated under diastolic conditions (80 mmHg and specimen-specific axial stretches) for all three genotypes: WT, C1041G, and mgR. Shown, too, are associated biaxial Cauchy stress (actual force per actual oriented area) – stretch curves for (B, E) ascending and (C, F) descending thoracic aorta, which reflect the material behavior at different levels of distension and extension. Data are shown as mean ± SEM, with **** $p < 0.0001$.

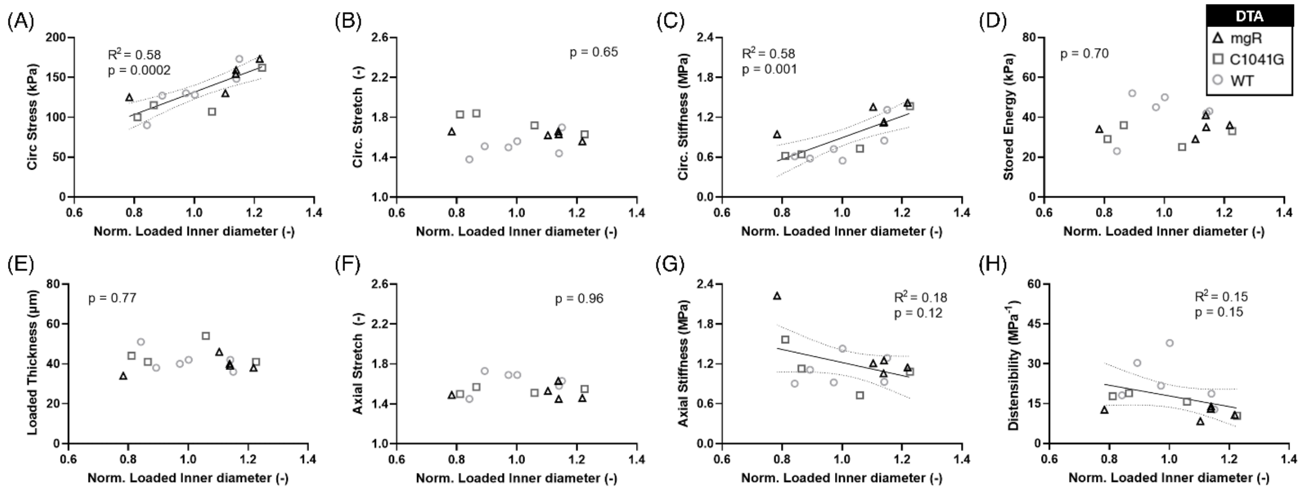

Supplementary Figure 5. Plots of specimen-specific geometric and mechanical metrics for the descending thoracic aorta (DTA) vs. normalized diameter, all calculated at in vivo relevant diastolic conditions of 80 mmHg and specimen-specific axial stretch, for each genotype: WT (*n*=6 - circles), C1041G (*n*=4 - squares), and mgR (*n*=5 - triangles). The data were fit by linear regression, with the best-fit line and 95% confidence intervals (solid and dotted lines, respectively) shown when the slope was nonzero or showed a nearly significant trend.

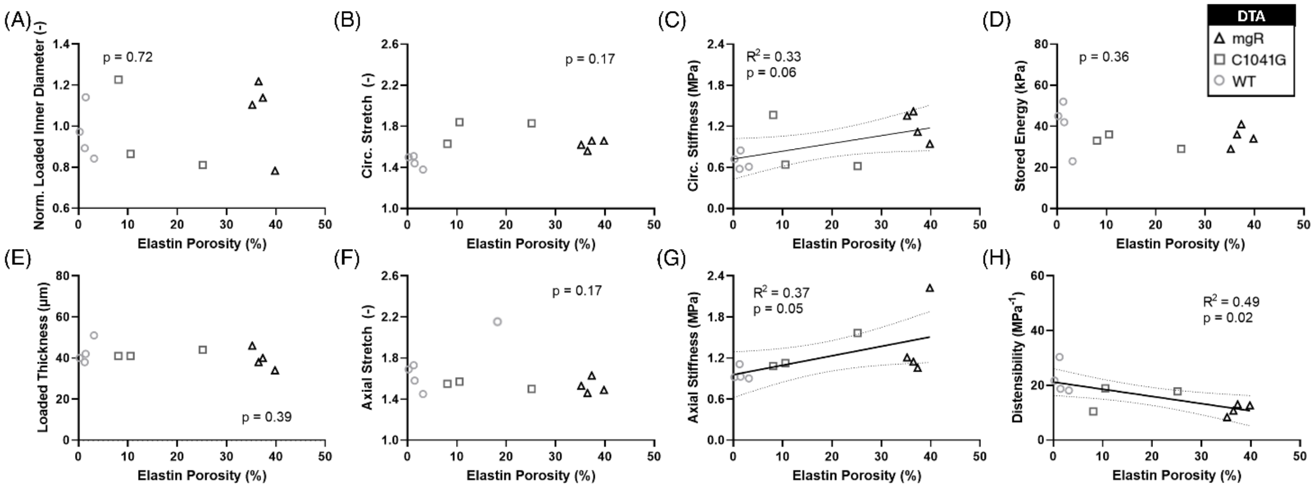

Supplementary Figure 6. Plots of specimen-specific geometric and mechanical metrics for the descending thoracic aorta (DTA) vs. elastin porosity, all calculated at in vivo relevant diastolic conditions of 80 mmHg and specimen-specific axial stretch, for each genotype: WT (*n*=4), C1041G (*n*=3), and mgR (*n*=4). The data were fit by linear regression, with the best-fit line and 95% confidence intervals (solid and dotted lines, respectively) shown when the slope was nonzero or showed a nearly significant trend.

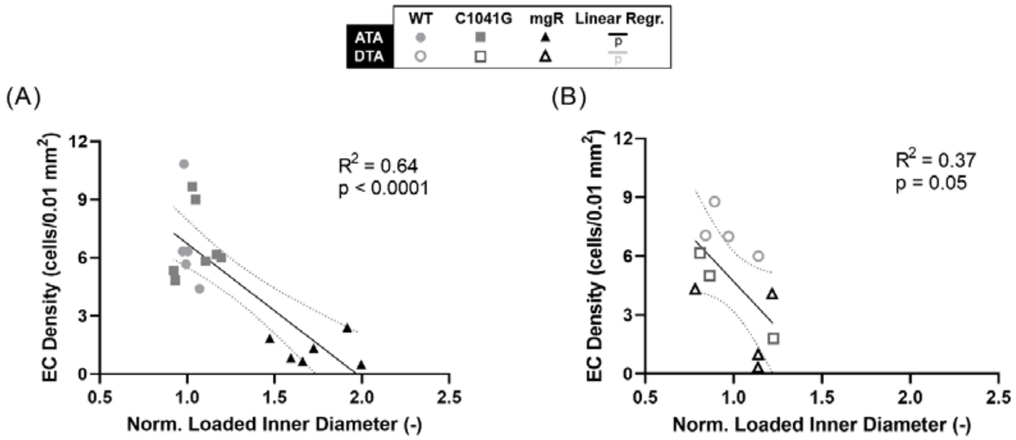

Supplementary Figure 7. Plots of specimen-specific endothelial cell density based on cell nuclei and associated areas vs. specimen-specific normalized inner diameter for (A) the ascending (ATA, solid markers) and (B) descending (DTA, open symbols) thoracic aorta, all calculated at in vivo relevant diastolic conditions of 80 mmHg and specimen-specific axial stretch for each genotype: WT (circles), C1041G (squares), and mgR (triangles). The data were fit by linear regression, with the best-fit line and 95% confidence intervals (solid and dotted lines, respectively).

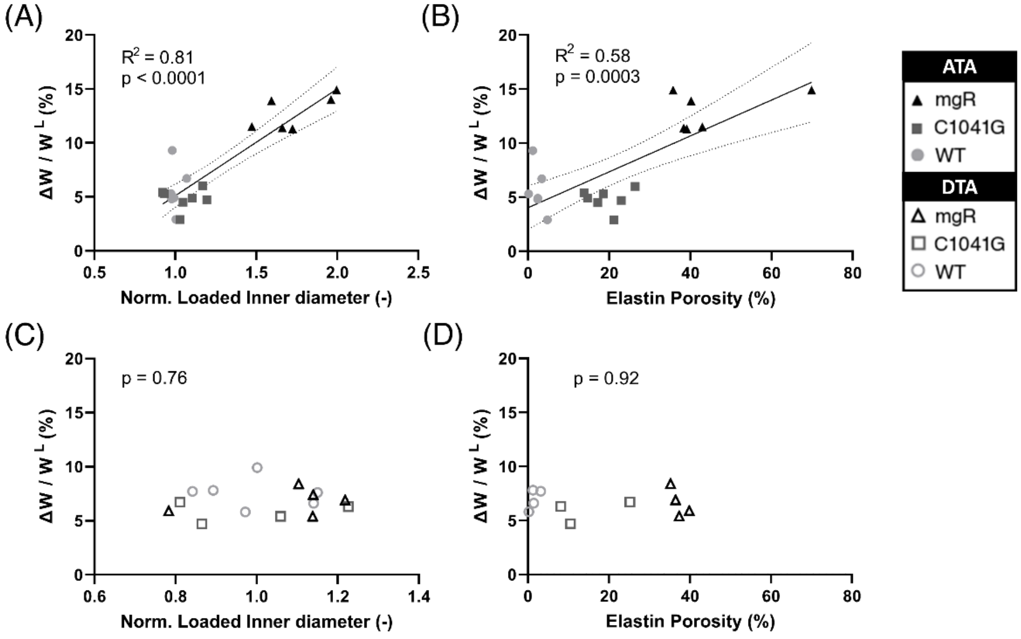

Supplementary Figure 8. Plots of specimen-specific energy dissipation (that is, energy lost during cyclic loading, inferred as the difference between elastic energy gained upon loading and elastic energy retained during unloading) vs. specimen-specific (A, B) diameter and (C, D) elastin porosity for the ascending (ATA) and descending (DTA) thoracic aorta for all three genotypes. The data were fit by linear regression, with the best-fit line and 95% confidence intervals (solid and dotted lines, respectively) shown when the slope was nonzero or showed a nearly significant trend.

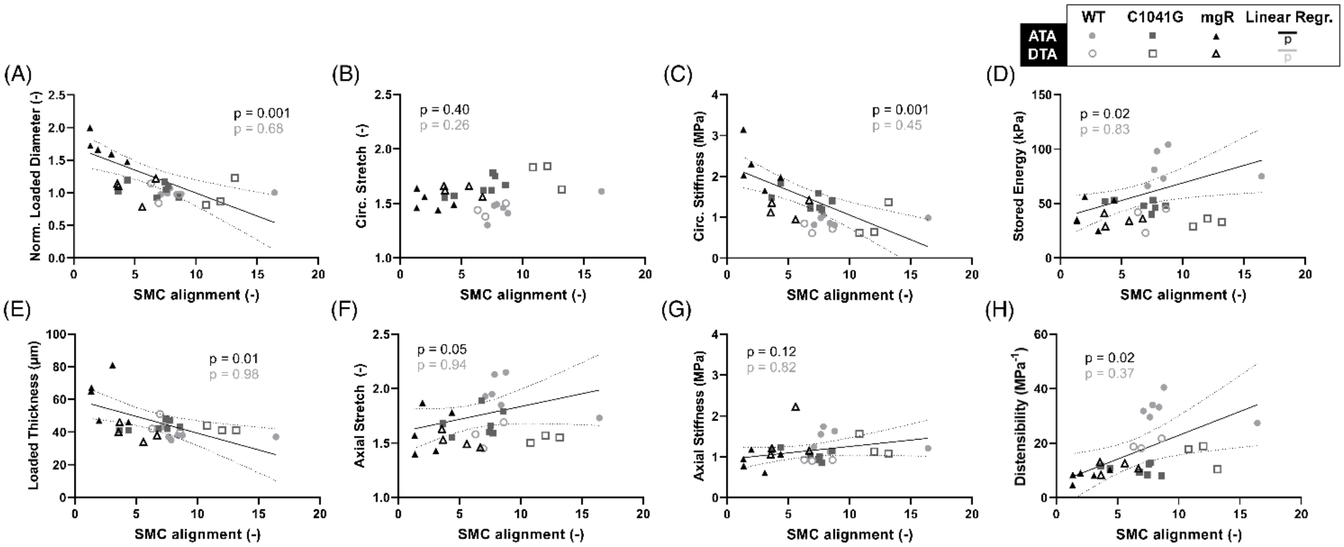

Supplementary Figure 9. Plots of specimen-specific geometric and mechanical metrics vs. smooth muscle cell alignment for the ascending (ATA, solid markers) and descending (DTA, open symbols) thoracic aorta. The data were fit by linear regression, with the best-fit line and 95% confidence intervals (solid and dotted lines, respectively) shown when the slope was nonzero or showed a nearly significant trend.

1.2 Supplementary Tables

Supplementary Table 1. Geometric and mechanical metrics for the three genotypes (WT, C1041G, mgR) for the ascending (ATA) and the descending (DTA) thoracic aorta. Values are mean ± SEM. Pressure values (P) are given in mmHg.

	**ATA – at Diastolic Pressure**			**DTA – at Diastolic Pressure**		
	**WT**	**C1041G**	**mgR**	**WT**	**C1041G**	**mgR**
	***n* = 6**	***n* = 7**	***n* = 6**	***n* = 6**	***n* = 4**	***n* = 5**
**Unloaded dimensions**						
Wall Thickness (μm)	107 ± 1.8	120 ± 4.6	146 ± 7.8	102 ± 2.2	120 ± 7.3	97 ± 5.9
Outer Diameter (μm)	1129 ± 28	1077 ± 40	1801 ± 101	800 ± 28	726 ± 72	796 ± 55
Axial Length (mm)	2.15 ± 0.14	2.73 ± 0.12	3.57 ± 0.10	3.54 ± 0.29	3.94 ± 0.60	4.95 ± 0.39
**Loaded dimensions**	**P = 80**	**P = 80**	**P = 80**	**P = 80**	**P = 80**	**P = 80**
Outer Diameter (μm)	1523 ± 19.9	1617 ± 59.2	2569 ± 130.8	1099 ± 50.1	1096 ± 97.4	1173 ± 79.1
Wall Thickness (μm)	38 ± 1.1	43 ± 1.1	61 ± 6.7	42 ± 2.1	45 ± 3.0	39 ± 1.9
Inner Radius (μm)	794 ± 12.1	794 ± 30.5	1256 ± 60.9	543 ± 26.2	528 ± 47.7	567 ± 39.7
In vivo Axial Stretch (λ_z^{iv})	1.96 ± 0.07	1.68 ± 0.05	1.61 ± 0.09	1.63 ± 0.04	1.53 ± 0.02	1.51 ± 0.03
In vivo Circumferential Stretch (λ_ϑ)	1.46 ± 0.04	1.65 ± 0.03	1.52 ± 0.04	1.51 ± 0.04	1.75 ± 0.05	1.63 ± 0.02
**Diastolic Cauchy Stresses (kPa)**						
Circumferential, σ_ϑ	206 ± 8.4	188 ± 7.4	222 ± 21.3	133 ± 11.1	121 ± 14.1	148 ± 9.0
Axial, σ_z	294 ± 20.3	187 ± 7.9	157 ± 22.4	165 ± 11.6	120 ± 9.4	131 ± 7.0
**Diastolic Linearized Stiffness (MPa)**						
Circumferential,C_{qqqq}	0.92 ± 0.04	1.42 ± 0.09	2.22 ± 0.25	0.77 ± 0.12	0.84 ± 0.18	1.19 ± 0.09
Axial, C_{zzzz}	1.45 ± 0.07	2.69 ± 0.36	3.85 ± 0.67	1.10 ± 0.90	1.13 ± 0.17	1.38 ± 0.21
**Diastolic Stored Energy (kPa)**	83 ± 6.1	49 ± 1.8	41 ± 6.0	42 ± 4.3	31 ± 2.4	35 ± 1.9
**Distensibility (1/MPa)**	32.76 ± 1.83	10.49 ± 0.73	8.09 ± 0.93	23.26 ± 3.75	15.72 ± 1.89	11.74 ± 0.99
**% $\Delta W / W^L$**	5.6 ± 0.9	4.8 ± 0.4	12.6 ± 0.8	6.6 ± 7.7	5.4 ± 6.7	5.4 ± 6.9

	**ATA – at Systolic Pressure**			**DTA – at Systolic Pressure**		
	**WT**	**C1041G**	**mgR**	**WT**	**C1041G**	**mgR**
	***n* = 6**	***n* = 7**	***n* = 6**	***n* = 6**	***n* = 4**	***n* = 5**
**Loaded dimensions**	**P = 120**	**P = 120**	**P = 120**	**P = 120**	**P = 120**	**P = 120**
Outer Diameter (μm)	1766 ± 27.1	1699 ± 63.8	2667 ± 124.0	1215 ± 51.4	1172 ± 95.9	1237 ± 83.1
Wall Thickness (μm)	32 ± 1.0	41 ± 1.1	59 ± 6.5	37 ± 2.0	42 ± 2.8	37 ± 2.0
Inner Radius (μm)	850 ± 14.3	808 ± 31.6	1275 ± 59.7	570 ± 26.8	544 ± 47.2	581 ± 40.6
In vivo Axial Stretch (λ_z^{iv})	1.96 ± 0.07	1.68 ± 0.05	1.61 ± 0.09	1.63 ± 0.04	1.53 ± 0.02	1.51 ± 0.03
In vivo Circumferential Stretch (λ_ϑ)	1.70 ± 0.04	1.74 ± 0.04	1.58 ± 0.04	1.69 ± 0.06	1.89 ± 0.07	1.72 ± 0.02
**Systolic Cauchy Stresses (kPa)**						
Circumferential, σ_ϑ	423 ± 18.7	315 ± 13.2	361 ± 35.5	249 ± 19.0	211 ± 20.7	250 ± 16.0
Axial, σ_z	395 ± 24.8	233 ± 10.1	206 ± 27.6	221 ± 17.8	165 ± 12.4	183 ± 7.5
**Systolic Linearized Stiffness (MPa)**						
Circumferential,C_{qqqq}	2.11 ± 0.10	5.42 ± 0.36	7.13 ± 1.31	1.71 ± 0.25	2.06 ± 0.51	2.68 ± 0.20
Axial, C_{zzzz}	2.06 ± 0.14	1.39 ± 0.09	1.29 ± 0.12	1.71 ± 0.18	1.70 ± 0.29	2.20 ± 0.40
**Systolic Stored Energy (kPa)**	129 ± 9.5	61 ± 2.6	52 ± 7.8	62 ± 7.2	42 ± 2.9	46 ± 3.0

Supplementary Table 2. Average best-fit material parameters for the four-fiber family constitutive model determined via nonlinear regression for the three genotypes (WT, C1041G, mgR) for the ascending (ATA) and descending (DTA) thoracic aorta. Data are presented as mean ± SEM values.

		**Elastic Fibers**	**Axial Collagen**		**Circ. Collagen + SMC**		**Symmetric Diagonal Collagen**			**Error**
		c (kPa)	c_1^1 (kPa)	c_2^1	c_1^2 (kPa)	c_2^2	$c_1^{3,4}$ (kPa)	$c_2^{3,4}$	a_o (deg)	*RMSE*
**ATA**	WT	20.39 ± 4.11	4.88 ± 2.11	0.26 ± 0.26	25.45 ± 4.23	2.90E-10 ± 2.74E-10	14.25 ± 1.87	0.16 ± 0.02	46.87 ± 0.76	0.102 ± 0.004
	C1041G	14.90 ± 2.94	6.51 ± 0.89	0.09 ± 0.03	3.10E-05 ± 2.54E-05	3.67 ± 0.33	7.63 ± 0.20	0.44 ± 0.07	50.92 ± 1.11	0.080 ± 0.003
	mgR	15.91 ± 1.84	1.97 ± 0.50	1.07 ± 0.75	3.34E-05 ± 2.08E-05	8.74 ± 1.23	9.35 ± 1.65	0.92 ± 0.17	54.86 ± 1.12	0.147 ± 0.012
**DTA**	WT	17.00 ± 4.16	11.59 ± 4.01	0.15 ± 0.10	15.99 ± 2.83	0.10 ± 0.04	4.40 ± 1.36	0.65 ± 0.07	41.08 ± 2.23	0.081 ± 0.002
	C1041G	11.05 ± 1.72	6.72 ± 2.69	0.77 ± 0.38	3.68 ± 1.32	0.90 ± 0.85	2.16 ± 1.15	0.83 ± 0.14	40.11 ± 3.93	0.091 ± 0.004
	mgR	21.19 ± 1.67	2.39 ± 2.03	1.67 ± 0.52	4.53 ± 1.78	0.29 ± 0.09	2.19 ± 1.30	1.44 ± 0.35	41.72 ± 2.87	0.082 ± 0.003

Supplementary Table 3. Sample-specific diameter and best-fit material parameters for the four-fiber family constitutive model determined via nonlinear regression for the three genotypes (WT, C1041G, mgR) for the ascending (ATA) and descending (DTA) thoracic aorta. Diameter values are reported at the unloaded configuration and at *in vivo* relevant diastolic conditions of 80 mmHg (the latter also normalized for the average diameter of the WT group).

			**Sample Diameter**			**Material Parameters**								
			**Unloaded Inner Diameter (µm)**	**Loaded Inner Diameter (µm)**	**Normalized Loaded Inner Diameter(-)**	**Elastic Fibers**	**Axial Collagen**		**Circ. Collagen + SMC**		**Symmetric Diagonal Collagen**			**Error**
						c (kPa)	c_1^1 (kPa)	c_2^1	c_1^2 (kPa)	c_2^2	$c_1^{3,4}$ (kPa)	$c_2^{3,4}$	a_o (deg)	*RMSE*
**ATA**	*WT*	*WT 1*	1030	1526	1.00	24.10	10.99	2.24E-14	12.51	1.40E-12	9.73	0.25	45.35	0.091
		WT 2	1146	1497	0.98	34.05	9.05E-12	1.56	28.61	1.66E-09	8.57	0.13	44.68	0.114
		WT 3	1222	1495	0.98	22.88	1.25E-08	0.01	41.55	7.79E-11	17.85	0.20	47.53	0.091
		WT 4	1177	1620	1.06	14.02	3.58	2.22E-14	30.98	2.22E-14	13.60	0.10	46.88	0.113
		WT 5	1102	1511	0.99	4.49	11.54	2.39E-14	19.82	2.39E-14	20.33	0.12	49.97	0.102
		WT 6	1097	1492	0.98	22.82	3.16	3.51E-14	19.21	2.22E-14	15.42	0.17	46.81	0.100
	C1041G	*C1041G 1*	1199	1787	1.17	13.44	4.96	2.28E-03	2.43E-07	5.10	7.64	0.56	50.23	0.096
		C1041G 2	1085	1571	1.03	21.74	5.50	0.13	2.47E-05	4.18	7.93	0.57	52.66	0.072
		C1041G 3	1220	1812	1.19	28.53	6.33	0.22	1.82E-04	3.16	7.68	0.76	50.39	0.075
		C1041G 4	1052	1602	1.05	11.44	9.49	0.03	4.97E-08	2.70	8.16	0.26	47.80	0.083
		C1041G 5	1064	1694	1.11	14.66	3.68	0.14	1.07E-07	2.85	7.62	0.34	47.01	0.084
		C1041G 6	975	1434	0.94	7.12	10.03	0.04	6.80E-06	3.42	6.53	0.32	53.25	0.076
		C1041G 7	962	1420	0.93	7.39	5.61	0.04	2.89E-06	4.27	7.80	0.28	55.08	0.074
	mgR	*mgR 1*	1718	2631	1.73	21.07	2.62	0.69	1.03E-11	6.76	4.65	1.03	49.74	0.177
		mgR 2	1707	2498	1.64	9.15	3.23	2.00E-06	2.21E-06	5.51	12.06	0.40	56.59	0.121
		mgR 3	1582	2224	1.46	13.67	2.26	5.09E-08	6.14E-09	9.87	15.10	0.50	55.99	0.104
		mgR 4	1917	2919	1.92	16.25	2.73	1.88E-08	9.81E-05	7.92	6.44	1.01	57.50	0.163
		mgR 5	1821	2469	1.62	20.67	0.96	0.99	1.93E-10	14.13	6.62	1.54	54.59	0.147
		mgR 6	2175	3021	1.98	14.66	0.00	4.73	1.00E-04	8.24	11.24	1.04	54.76	0.173
**DTA**	*WT*	*WT 1*	928	1243	1.13	31.47	1.42	0.54	12.91	0.24	4.58	0.84	45.48	0.085
		WT 2	789	1069	0.97	14.56	15.90	2.32E-14	20.45	2.33E-14	2.96	0.59	46.63	0.075
		WT 3	759	957	0.87	0.00	28.09	3.66E-14	25.20	0.13	10.92	0.84	40.81	0.079
		WT 4	726	984	0.89	20.61	10.75	2.34E-14	15.38	2.34E-14	3.12	0.46	40.47	0.076
		WT 5	792	1103	1.00	17.12	11.12	2.22E-14	17.18	2.45E-14	3.25	0.45	31.17	0.082
	C1041G	*C1041G 1*	796	1184	1.08	8.89	4.62	0.37	4.02	0.12	2.42	0.66	40.22	0.092
		C1041G 2	895	1329	1.21	16.04	0.10	1.91	2.33E-14	3.44	5.30	0.66	50.22	0.099
		C1041G 3	595	912	0.83	8.67	11.64	0.51	4.46	0.06	0.45	1.24	31.08	0.081
		C1041G 4	618	961	0.87	10.60	10.53	0.30	6.23	1.26E-12	0.46	0.78	38.90	0.092
	mgR	*mgR 1*	831	1238	1.13	22.94	0.27	0.80	2.54	0.34	1.58	0.76	42.62	0.084
		mgR 2	826	1236	1.12	17.78	0.42	2.43	2.46	0.33	7.27	0.70	42.54	0.086
		mgR 3	904	1315	1.20	26.73	0.06	3.34	11.03	1.10E-12	1.51	1.57	46.96	0.071
		mgR 4	585	864	0.79	18.05	10.49	0.60	5.55	0.25	0.08	2.60	30.78	0.086
		mgR 5	835	1214	1.10	20.46	0.71	1.21	1.06	0.54	0.51	1.54	45.67	0.081
